# Towards risk-targeted seismic hazard models for Europe

**DOI:** 10.1038/s41598-023-36947-y

**Published:** 2023-07-03

**Authors:** Giorgio Monti, Cristoforo Demartino, Paolo Gardoni

**Affiliations:** 1 College of Civil Engineering and Architecture, Zhejiang University, 866 Yuhangtang Road, Hangzhou, 310058 Zhejiang People’s Republic of China; 2 Department of Structural Engineering and Geotechnics, Sapienza University of Rome, Via A. Gramsci 53, 00197 Rome, Italy; 3 Zhejiang University - University of Illinois at Urbana Champaign Institute, Zhejiang University, 718 East Haizhou Road, Haining, 314400 Zhejiang People’s Republic of China; 4 Department of Civil and Environmental Engineering, University of Illinois at Urbana-Champaign, Urbana, IL 61801 USA

**Keywords:** Civil engineering, Natural hazards

## Abstract

Standards and Codes of Practice for designing new constructions and for assessing and strengthening existing ones are usually based on uniform hazard maps, where different Limit States (*LS*s) are associated with different hazard-exceedance probabilities. This approach yields non-homogeneous *LS*-exceedance probabilities across a territory, thus failing to achieve the goal of uniform risk throughout a territory. Such lack of uniformity stems from estimating the probability of failure using capacity and demand models. If the capacity of new constructions—or the capacity increase of strengthened existing constructions—are designed based on a prescribed hazard-exceedance probability, then the seismic risk depends on both the structure (depending on the design philosophy and corresponding design objectives), through the capacity model, and the location, through the hazard model. The aim of this study is threefold. First, it provides a seismic probability assessment formulation and a risk-targeted intensity measure based on a linear model in log–log coordinates of the hazard, under the assumption of log-normal capacity and demand. The proposed framework introduces a factor that multiplies the code hazard-based demand to account either for intentional (from design) over-capacity or for undesired (e.g., in existing constructions) under-capacity. Second, this paper shows an application to peak ground accelerations in Europe considering parameters taken from Standards and Codes of Practice. The developed framework is used to determine the risk-target levels of peak ground acceleration used for design in Europe, for both new and existing constructions. Third, the obtained target risk levels are used to determine a risk-based intensity modification factor and a risk-based mean return period modification factor, which can be readily implemented in current Standards to achieve risk-targeted design actions, with equal *LS*-exceedance probability across the territory. The framework is independent of the chosen hazard-based intensity measure, be it the commonly used peak ground acceleration or any other measure. The results highlight that in large areas of Europe the design peak ground acceleration should be increased to achieve the proposed seismic risk target and that this is particularly significant for existing constructions, given their larger uncertainties and typical low capacity with respect to the code hazard-based demand.

## Introduction

The most common approach used nowadays to determine seismic-design loads for structural design is the Probabilistic Seismic-Hazard Analysis (PSHA)^1–4^. The intensity measure to be used in design is generally considering additional factors defining the local response (e.g., soil factors), and design procedures (e.g., importance and behavior factors). The primary output from a PSHA is a hazard curve associating exceedance rates (or its reciprocal, the return period) to different values of a selected intensity measure (i.e., a ground-motion parameter). The value of the intensity measure used in design is generally taken as that corresponding to a predefined return period. This approach is generally called uniform-hazard, because the seismic intensity used in design is obtained throughout a territory using the same annual frequency of exceedance. Current seismic building codes (e.g., Eurocode 8^5^) generally adopt such approach to define the intensity measure used for design. This has three principal advantages^6^: transparency, a uniform hazard level across a territory, and the ability to compare (and ideally control) risk for different types of hazard (e.g., earthquake and wind).

By integrating such hazard, considered either as linear in log-log coordinates^7–9^ or as a more complex function^10–12^, with a probabilistic description of the capacity, the Limit State (*LS*)-exceedance rate—the risk—is obtained. The resulting outcome of such approach has a well-known drawback in that it yields non-homogeneous *LS*-exceedance rates across a territory^13–16^, thus failing to achieve the goal of uniform risk. The main reason for this is related to the fact that, if the capacity of new constructions - or the capacity increase of strengthened existing constructions - (i.e., fragility) is defined based on a single prescribed hazard-exceedance probability, then the seismic risk - which depends on the whole spectrum of the hazard function - will present a site-to-site variability. The uniform-hazard approach provides consistent results only if both (*i*) capacity and demand models have no uncertainty, and (*ii*) the median capacity and the prescribed median design demand coincide [as it will be clearly demonstrated in Section “Explanation of non-uniform risk conditions” in Eq. (11)]. However, this is not the case, since uncertainty is in fact present in both capacity and demand models. It is noteworthy that non-homogeneous *LS*-exceedance can also be induced by the use of design spectra obtained by anchoring the predictive spectral shape to the peak ground acceleration^17,18^.

To overcome this drawback, different studies (e.g.,^6,19–21^) proposed design seismic actions based on “risk-targeting”, that is, aiming at obtaining the same annual LS-exceedance rates over an entire territory. Such method has the additional merit of using all the information contained in a hazard curve at a given site, as opposed to a uniform-hazard method, which only uses one value pertaining to the prescribed hazard-exceedance rate^20^.

The idea of using intensity measure values for a design that targets a specified seismic risk level was pioneered in ASCE/SEI 43-05^19^, a design standard for nuclear power plants. Two probability goals are provided to define this level of conservatism^22^: (*i*) less than about a 1% probability of unacceptable performance for the design basis earthquake (DBE) ground motion and (*ii*) less than about a 10% probability of unacceptable performance for a ground motion equal to 150% of the DBE ground motion. The “seismic capacity point” is defined on a lognormal fragility curve in terms of the median capacity and logarithmic standard deviation. A 1% fractile was employed for the capacity^7^.

An explicit probabilistic approach, which allows for the explicit quantification of the exceedance probability of different limit states, has not yet been implemented in building seismic codes^23^, except for the ASCE-7 standard^24^, FEMA^25^ and Indonesian codes^26^. The first study proposing a risk-targeted design maps for the U.S. was in 2007 by Luco et al.^13^ where the fragility model of the collapse limit state was described by a lognormal distribution parameterized with the 10th-percentile collapse capacity and a log-standard deviation taken as 0.8. Luco et al.^13^ adopted an acceptable national risk of 1% in 50 years corresponding to a mean annual collapse rate of $2\times 10^{-4}$ for the conterminous United States. ASCE 7-16^24^ uses this recommended value to provide risk-targeted seismic maps, where risk-targeted modification factors ranging from around 0.7 to 1.15 are applied to the Maximum Considered Earthquake ground motions. Luco et al.^13^ and FEMA P750^27^ adopted a log-standard deviation of the lognormal fragility curves equal to 0.8, while Chapter 21 of ASCE 7-16^24^ specifies 0.6. The informative annex of the new draft of Eurocode 8^28^ proposes a target annual occurrence rate of $2\times 10^{-4}$ for the near-collapse *LS* for consequence class 2. It is noteworthy that different authors observed that larger log-standard deviations in regions with high seismic hazard lead to “unrealistically large collapse probabilities”^29,30^.

Currently, no meaningful reference can be found in European design codes or guidelines, but some relevant surveys have been conducted in recent years, and various annual collapse probabilities have been proposed. Recently, the new draft of Eurocode 8^28^ includes an informative annex for a simplified reliability-based verification format. Nevertheless, different authors proposed risk-targeted maps for France^29^, Italy^31–33^, Spain^34^, Romania^35^, Iran^36–38^, Indonesia^39,40^, China^41^, Korea^42^ and Europe^14,16^. We refer the interested readers to^6,20^ for a review of the seismic risk-targeted approaches. The aforementioned studies mainly focused on the collapse *LS* and employed variable log-standard deviation of the fragility curves (ranging from 0.3 to 0.4) and different target *LS*-exceedance probabilities, i.e., risk targets. Besides, different studies tried to calculate the seismic risk implied by current design standards^16,43–46^. It is noteworthy that several codes adopt reliability-targeted loads in other (non-seismic) contexts (e.g.,^47^). Gkimprixis et al.^16^ compared three seismic design approaches for a benchmark 4-storey 3-bay RC frame building across different regions in Europe. The findings reveal that the risk-targeted approach provides a means for directly controlling seismic risk, while the uniform-hazard approach exhibits constraints in achieving uniform risk levels.

Risk-targeted approaches are promising and, after some careful calibration, they are finding their way into the next generation of design codes in Europe^46^, even though still at the informative level. Accordingly, the development of a seismic risk-targeted model for Europe will play a central role to guarantee comparable safety conditions across a territory characterized by a strongly variable seismicity. However, although the problem of seismic risk target has been investigated in several studies, little attention has been paid to the selection of an appropriate code-compliant seismic target risk for the different *LS*s and the implementation of risk-based modification factors.

Along these lines, the aim of this study is threefold. First, it provides a unified probability-based formulation that applies to, both, design and assessment/strengthening. Second, it shows an application to peak ground accelerations in Europe, considering parameters taken from Standards and Codes of Practice. Third, it proposes a seismic risk-targeted model for Europe by defining seismic target-risk and risk-based modification factors. These factors can be readily implemented in current codes to obtain risk-targeted actions, for design, assessment, and strengthening, with equal *LS*-exceedance probability across a territory, without the need of changing the current hazard-based maps. As an example, the proposed approach is applied to the European territory. The framework is independent of the chosen hazard-based intensity measure, be it the commonly used peak ground acceleration or any other measure.

This paper is organized as follows. Section “Seismic probability assessment formulation” provides a seismic risk assessment formulation and a risk-targeted criterion based on a linear model in log-log coordinates of the hazard, under the assumption of log-normal capacity and demand. The proposed framework introduces a factor that shifts the median capacity, with respect to the code hazard-based demand, to account either for intentional (from design) over-capacity or for undesired (e.g., in existing constructions) under-capacity. Section “Risk-targeted intensity measure” discusses the definition of a risk-targeted intensity measure (or return period) and defines a risk-based intensity (or return period) modification factor, which can be readily implemented in current Standards to achieve risk-targeted design actions, with equal *LS*-exceedance probability across the territory. Section “Application to Europe for peak ground accelerations” provides an application to Europe for peak ground accelerations. The previously presented model is used to determine the risk-target levels for Europe for both new and existing constructions. Finally, in Section “Conclusions”, some conclusions are drawn about the implications for future seismic risk-targeted policies and studies in Europe.

## Seismic probability assessment formulation

In a structural system subjected to a certain hazard described by a function $\lambda (im)$, representing the mean annual frequency of exceeding a given intensity level *im*, the mean annual exceedance frequency $\lambda _{f,LS}$ of a specified *LS*, is found as (e.g.,^9^):

1 $$\begin{aligned} \lambda _{f,LS}=\intop _{\Omega _{im}}F_{LS}(im)\cdot \left| \mathrm {d\lambda }(im)\right| \end{aligned}$$

where $\Omega _{im}$ is the domain of *im*. The term “mean”, here and elsewhere, refers to the mean estimate of this frequency^48^.

In Eq. (1), $F_{LS}(im)$ is the so-called fragility function relevant to the *LS* of interest, defined as:

2 $$\begin{aligned} F_{LS}(im)=P(D\ge C_{LS}|im) \end{aligned}$$

where *D* is the demand on the system and $C_{LS}$ is the capacity of the system at the specified *LS*. In the general case, $D(\textbf{x},im)$ and $C_{LS}(\textbf{x},im)$ depend on both the system properties vector $\textbf{x}$ and the hazard intensity measure *im*, and are expressed in the form of Engineering Demand Parameters (*EDP*s). These are practical structural response quantities used to estimate damage to structural and nonstructural components and systems. In order to compare capacity and demand, the same *EDP*s must be used, with an exceedance criterion depending on the considered *LS*. Different *EDP*s can however be used for different *LS*s. The transformation from the hazard intensity measure into an *EDP* is generally obtained through a structural model.

In the following, a probability formulation based on a linear hazard model in log-log coordinates, under the assumption of log-normal capacity and demand, is provided. The introduced simplifications allow developing a closed-form formulation to facilitate its application in code-based seismic engineering design practices.

### Hazard and demand models

Both the hazard and the demand model are defined through functions expressing their median. The associated uncertainty is expediently included at a later stage.

The median hazard function in Eq. (1) can be assumed as^8,9^:

3 $$\begin{aligned} \lambda (im)=k_{0}im^{-k_{1}} \end{aligned}$$

which is a linear curve in the log-log plane, i.e., $\ln \lambda (im)=\ln k_{0}-k_{1}\ln im$, with $k_{0}>0$ and $k_{1}>0$ being site-specific purely numerical constants. The log-linear model is adopted to obtain a closed form solution of the proposed framework. Several non-linear models are available in the literature (see Section “Introduction”).

Demand is considered as a lognormally distributed random variable of the chosen *EDP*. The median conditioned on the value of *im* can be approximated as^9,49^:

4 $$\begin{aligned} \hat{D}(im)=a\cdot im^{b} \end{aligned}$$

where *a* and *b* are two constants to be determined through numerical nonlinear analyses. *b* describes the non-linear relationship between the median *EDP* and *im* and is generally larger than 1 for structures with short fundamental period (e.g.,^50,51^). The so-called “equal displacement rule”^52^ suggests that the relationship between the median inelastic displacements (*EDP*) and *im* may be approximately linear, thus implying $b=1$. This common assumption is valid for both buildings^53^ and bridges^53,54^. In the following analytical developments, *b* will be retained to show its role in the resulting equations.

### Capacity model

The capacity of the structural system is either designed for new constructions or assessed for existing constructions. Current seismic building codes generally adopt a constant hazard approach, where the intensity used to check a given *LS* has the same exceedance frequency throughout the territory. For any *LS* considered, the seismic action used in design is given in terms of its return period $T_{R,LS}$, from which its mean annual frequency can be obtained as:

5 $$\begin{aligned} \lambda _{LS}=\frac{1}{T_{R,LS}} \end{aligned}$$

The return period $T_{R,LS}$ is usually obtained by fixing the hazard-exceedance probability $P_{V_{R},LS}$ within a specified reference period $V_{R}$ (for example, 10% in 50 years for Life Safety *LS*, which results in $T_{R,LS}=-V_{R}/\ln (1-P_{V_{R},LS})\approx 475$ years). The corresponding hazard-based median intensity, $\hat{im}_{LS,haz}$, is the intensity measure corresponding to $\lambda _{LS}$ obtained by inverting Eq. (3):

6 $$\begin{aligned} \hat{im}_{LS,haz}=\left( \frac{k_{0}}{\lambda _{LS}}\right) ^{\frac{1}{k_{1}}} \end{aligned}$$

Substituting Eq. (6) into Eq. (4), the median demand in terms of the chosen *EDP* is calculated. Seismically designed structures in general have a larger median capacity than that required by the median demand, while for existing structures the opposite is generally true due, for instance, to degradation and/or design with less demanding Codes. Thus, the median *EDP* capacity is defined as the median *EDP* demand multiplied by a factor $\gamma _{R,LS}$ (see, for e.g.,^19,55^ and^7,56–59^):

7 $$\begin{aligned} \hat{C}_{LS}=\gamma _{R,LS}\cdot a\cdot \hat{im}_{LS,haz}^{b} \end{aligned}$$

Such factor $\gamma _{R,LS}$ accounts for over-strength and ductility either for intentional (from design) over-capacity ($>1$) or undesired (e.g., in existing constructions) under-capacity ($<1$) with respect to the code hazard-based median *EDP* demand pertaining to a certain *LS*. A practical interpretation will be given in Section “Calibration of the seismic target risk for new constructions”.

### Log-normality assumption for capacity and demand

The EDPs for demand and capacity can be assumed as log-normally distributed and independent (e.g.,^9,11,12,60^). Under this assumption, the fragility function is also log-normal (e.g.,^61^) and can be obtained using Eqs. (4) and (7) as:

8 $$\begin{aligned} F_{LS}(im)=\Phi \left[ \frac{1}{\beta _{LS}}\ln \left( \frac{\hat{D}(im)}{\hat{C}_{LS}}\right) \right] =\Phi \left[ \frac{1}{\beta _{LS}}\ln \left( \frac{im^{b}}{\gamma _{R,LS}\cdot \hat{im}_{LS,haz}^{b}}\right) \right] \end{aligned}$$

where $\Phi \left[ \bullet \right]$ is the standard normal cumulative distribution, $\hat{D}(im)$ is the median of the *EDP* demand conditioned on the value of *im* [Eq. (4)], $\hat{C}_{LS}$ is the median of the capacity [Eq. (7)], and $\beta _{LS}$ is the log-standard deviation of the safety margin in terms of natural logarithms $\ln D-\ln C$ defined as:

9 $$\begin{aligned} \beta _{LS}=\sqrt{\beta _{D,LS}^{2}+\beta _{C,LS}^{2}} \end{aligned}$$

where $\beta _{D,LS}$ and $\beta _{C,LS}$ are the log-standard deviation of the *EDP* demand and capacity, respectively. It is noteworthy that Eq. (8) does not depend on *a* [Eq. (7)], and that if $b=1$ then *EDP* and *im* can be used indifferently (while however retaining the *EDP* uncertainties).

### Calculation of $\lambda _{f,LS}$

Using Eqs. (3) and (8), it can be observed that Eq. (1) is equivalent to^7–9,62^:

10 $$\begin{aligned} \lambda _{f,LS}=\frac{1}{\gamma _{R,LS}^{\frac{k_{1}}{b}}}\cdot \lambda _{LS}\cdot \exp \left( \frac{1}{2}\frac{k_{1}^{2}}{b^{2}}\beta _{LS}^{2}\right) \end{aligned}$$

where it can be observed that $\lambda _{f,LS}$ is equal to the annual exceedance frequency $\lambda _{LS}$ of the hazard-based intensity $\hat{im}_{LS,haz}$, increased by a term depending on both the hazard slope $k_{1}$ and the dispersion $\beta _{LS}$. As for the role of the factor $\gamma _{R,LS}$, Eq. (10) shows that any modification to the median capacity through the factor $\gamma _{R,LS}$ is affected by the coefficient *b* and, more importantly, by the slope of the hazard $k_{1}$. In a sense, this confirms that an over-capacity $\gamma _{R,LS}>1$ obtained following the indication of the code results in different risk depending on the local hazard slope. This issue will be discussed in the next section.

### Explanation of non-uniform risk conditions

**Figure 1:**
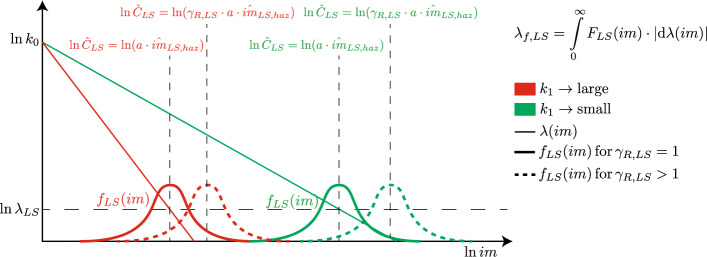
Schematic of the different elements for calculating the mean annual Limit State (*LS*)-exceedance frequency, $\lambda _{f,LS}$ ($b=1$). $f_{LS}(im)$ denotes the PDF of the fragility function in Eq. (8).

The schematic of the different elements for calculating $\lambda _{f,LS}$ [Eq. (10)] is reported in Fig. 1. The hazard curves, $\lambda (im)$, and the PDF function of the fragility curve, $f_{LS}(im)$, are reported in the log-log plane where the abscissa is $\ln im$. Two different hazard curves [Eq. (3)] with same $k_{0}$ but different $k_{1}$, large (thin solid red line) and small (thin solid green line), are considered as representative of two different sites. In both cases, the medians of the EDP capacity are first designed using Eq. (7) with $\gamma _{R,LS}=1$ (thick solid lines) assuming a certain value of $\lambda _{LS}$; a generic $\beta _{LS}\ge 0$ is assumed. The fragility curves are also shown shifted by $\gamma _{R,LS}>1$ (thick dashed lines), representing over-capacity (case of new constructions).

From Fig. 1, it can be seen that the solution of Eq. (10) depends on $k_{1}$ (local site seismicity) and reveals the following three cases:

11 $$\begin{aligned} \lambda _{f,LS}={\left\{ \begin{array}{ll} \frac{1}{\gamma _{R,LS}^{\frac{k_{1}}{b}}}\cdot \lambda _{LS}\cdot \exp \left( \frac{1}{2}\frac{k_{1}^{2}}{b^{2}}\beta _{LS}^{2}\right) &{} \text{if}\,\beta _{LS}>0\,\text{and}\,\gamma _{R,LS}\ne 1\\ \frac{1}{\gamma _{R,LS}^{\frac{k_{1}}{b}}}\cdot \lambda _{LS} &{} \text{if}\,\beta _{LS}=0\,\text{and}\,\gamma _{R,LS}\ne 1\\ \lambda _{LS} &{} \text{if}\,\beta _{LS}=0\,\text{and}\,\gamma _{R,LS}=1 \end{array}\right. } \end{aligned}$$

In the first case, $\lambda _{f,LS}$ is different from $\lambda _{LS}$ and the solution depends on $\beta _{LS}$, *b*, $\gamma _{R,LS}$, and $k_{1}$. Being $k_{1}$ a site-dependent regional constant (see Fig. 1), non-uniform risk ensues^13^. In the second case, the solution depends on $\gamma _{R,LS}$, *b*, and $k_{1}$, thus non-uniform risk ensues. Only when $\beta _{LS}=0$ and $\gamma _{R,LS}=1$, uniform risk ensues, with $\lambda _{f,LS}$ equal to $\lambda _{LS}$.

It can be concluded that only for the deterministic case ($\beta _{LS}=0$) with no over-capacity ($\gamma _{R,LS}=1$), the traditional design approach using hazard with uniform exceedance probability (see Section “Capacity model”) leads to uniform risk conditions. However, the case $\beta _{LS}=0$ and $\gamma _{R,LS}=1$ is a theoretical one, because uncertainties and over-capacity are always present; therefore, the real case results in uncontrolled values of $\lambda _{f,LS}$ depending on the structure ($\beta _{LS}$, $\gamma _{R,LS}$, *b*) and the location ($k_{1}$).

## Risk-targeted intensity measure

The aim of risk-targeting approaches is to control the risk of exceeding a given *LS* related to an unsatisfactory performance of the structure^21^. Section “Explanation of non-uniform risk conditions” demonstrated that the traditional design approaches based on constant hazard (Section “Capacity model”) defined in terms of $\lambda _{LS}$ results in different values of the *LS*-exceedance probability throughout a territory.

Probabilistic reliability methods are based on the comparison of the annual *LS*-exceedance probability $P_{f1,LS}$ with a target (acceptable) value $\widetilde{P}_{f1,LS}$. Design methods are calibrated in such a way that $P_{f1,LS}\approx \widetilde{P}_{f1,LS}$. For instance, in Eurocode 0^63^ and ISO 2394^64^, reliability requirements are expressed in terms of probability and are related to expected social and economic consequences (e.g.,^65^). However, to the author’s knowledge, target probability values specific to seismic design, whose annual values can be denoted as $\widetilde{P}_{f1,LS,seis}$, are still not provided by any Standard or Code of Practice, as stated in Section “Introduction”. It should be expected that target probabilities for seismic design be larger than those accepted for non-seismic design, that is, $\widetilde{P}_{f1,LS,seis}>\widetilde{P}_{f1,LS}$^66^. In the following, since results will be given in terms of frequency, the risk-targeted mean annual frequency for seismic design $\widetilde{\lambda }_{f1,LS,seis}$ will be adopted. For simplicity, it will be referred to as $\widetilde{\lambda }_{f,LS}$. Under a Poissonian assumption, notice that frequency is related to probability through $\lambda =-\ln (1-P)$, and that, when lower than $10^{-2}$, they can be used interchangeably for practical purposes, i.e., $\lambda \approx P$.

The risk-targeted mean annual frequency of hazard exceedance $\lambda _{LS,risk}$ can be obtained by replacing $\lambda _{LS}$ with $\widetilde{\lambda }_{f,LS}$ into Eq. (10) and solving to obtain:

12 $$\begin{aligned} \lambda _{LS,risk}=\gamma _{R,LS}^{\frac{k_{1}}{b}}\cdot \widetilde{\lambda }_{f,LS}\cdot \exp \left( -\frac{1}{2}\frac{k_{1}^{2}}{b^{2}}\beta _{LS}^{2}\right) \end{aligned}$$

The corresponding risk-targeted intensity $\hat{im}_{d,LS,risk}$ is obtained by replacing $\lambda _{LS,risk}$ for $\lambda _{LS}$ into Eq. (6). In order to find $\lambda _{LS,risk}$, the seismic target $\widetilde{\lambda }_{f,LS}$ should be set. This will be the object of the following section.

### Target risk

In this study, the minimum target annual *LS*-exceedance frequency ($\widetilde{\lambda }_{f,LS}$) across a territory is proposed as target risk. The basic idea behind this is to try to make constructions as safe as the safest one across a territory. As previously discussed, the definition of the target risk is related to expected social and economic consequences and, as such, different definitions are possible (e.g.,^30,67^). The proposed approach is implicitly considering the latter elements in the calibration by finding the target risk obtained with a design based on a code.

#### New constructions

A possible way to determine $\widetilde{\lambda }_{f,LS}$ is to use Eq. (10) to obtain the annual *LS*-exceedance frequency $\lambda _{f,LS}$ of constructions subjected to a hazard-based intensity $\hat{im}_{LS,haz}$ corresponding to a specified $\lambda _{LS}$.

For given values of $\lambda _{LS}$ and of the parameters ($\gamma _{R,LS}$, *b*, $\beta _{LS}$), Eq. (10) has a certain distribution throughout a territory as a function of $k_{1}$. The seismic target annual *LS*-exceedance frequency relevant to that territory can be evaluated as a certain fractile of such distribution. In this study, it is proposed to define it as the minimum value in this distribution:

13 $$\begin{aligned} \widetilde{\lambda }_{f,LS}=\min _{k_{1}}\left[ \frac{1}{\gamma _{R,LS}^{\frac{k_{1}}{b}}}\cdot \lambda _{LS}\cdot \exp \left( \frac{1}{2}\frac{k_{1}^{2}}{b^{2}}\beta _{LS}^{2}\right) \right] \end{aligned}$$

where the functions “$\min$” is intended “over the territory of interest”, whose hazard characteristics are expressed in terms of $k_{1}$. As a consequence of this choice, all constructions will be designed with the same target risk corresponding to the safest construction set of the considered population, characterized by the set of parameters ($\gamma _{R,LS}$, *b*, $\beta _{LS}$).

In selecting the safest construction, one should consider the following fundamental rationale. The median lateral capacity of a new construction is the maximum one resulting from design against, both, seismic and non-seismic (e.g., vertical loads, etc.) actions:

14 $$\begin{aligned} \hat{C}_{LS}=\max \left\{ \gamma _{R,LS}\cdot a\cdot \hat{im}_{LS,haz}^{b},\hat{C}_{NS,LS}\right\} \end{aligned}$$

where $\hat{C}_{NS,LS}$ is the median lateral capacity obtained from non-seismic actions. The first term in $\left\{ \cdot \right\}$ represents cases where the seismic action dominates design, while the second term where non-seismic actions dominate. The second case is typical of areas with relatively low seismicity, implying that the design capacity of new constructions is larger than that obtained from seismic actions, which leads to much lower *LS*-exceedance frequencies (e.g.,^68^). Therefore, if one considered Eq. (14) to define $\hat{C}_{LS}$ , the minimum of $\widetilde{\lambda }_{f,LS}$ in Eq. (13) across a territory will be dominated by constructions located in areas with low seismicity. This would result in the undesired consequence that the target risk be that of constructions designed for non-seismic loads. This would render the design of constructions in seismic areas uselessly conservative. Consequently, in the following, the median capacity considered is that obtained from design against seismic action.

#### Existing constructions: upgrade levels

Prediction of *LS*-exceedance frequency for existing structures under seismic loads has always been a crucial aspect of earthquake engineering. The assessed seismic median capacity of existing constructions, $\hat{C}_{LS,ass}$, is generally estimated by performing non-linear analyses using the mean values of measurable basic (geometric and mechanical) variables. The seismic median capacity obtained from assessing an existing construction can be expressed as [see also Eq. (7)]:

15 $$\begin{aligned} \hat{C}_{LS,ass}=\gamma _{R,LS,ass}\cdot a\cdot \hat{im}_{LS,haz}^{b} \end{aligned}$$

where $\gamma _{R,LS,ass}$ is the factor representing the ratio between the seismic median capacity of the existing construction and the seismic median *EDP* corresponding to the median hazard-based seismic intensity used in design, $\hat{im}_{LS,haz}$.

**Figure 2:**
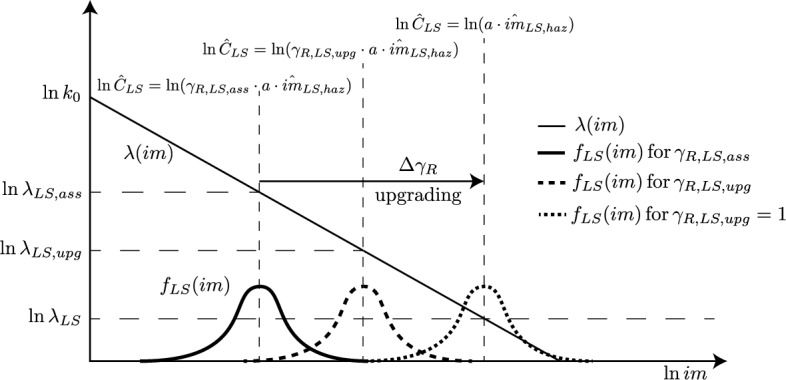
Schematic of the seismic risk assessment of an existing construction ($b=1$), with two upgrade strategies: “retrofit”, which uses the same action level as for design, and “upgrade”, which aims at a lower action level.

Figure 2 shows the schematic of an existing construction having a median capacity lower than the corresponding demand, i.e., $\gamma _{R,LS,ass}<1$, which implies $\lambda _{LS,ass}>\lambda _{LS}$. Consequently, a seismic upgrade is needed, which requires to modify the existing structure to increase its seismic capacity, so that:

16 $$\begin{aligned} \hat{C}_{LS,upg}=\gamma _{R,LS,upg}\cdot a\cdot \hat{im}_{LS,haz}^{b} \end{aligned}$$

with $\gamma _{R,LS,upg}=\gamma _{R,LS,ass}+\Delta \gamma _{R,LS}$, with $\Delta \gamma _{R,LS}>0$. It is important to note that *b* in Eqs. (15) and (16) can be different.

Two possible upgrade strategies are shown in the figure: (1) a full upgrade, denoted as “retrofit”, i.e., $\gamma _{R,LS,upg}=1$, which implies $\lambda _{LS,upg}=\lambda _{LS}$, and (2) a partial upgrade, i.e., $\gamma _{R,LS,ass}<\gamma _{R,LS,upg}<1$, which implies $\lambda _{LS,ass}>\lambda _{LS,upg}>\lambda _{LS}$. $\gamma _{R,LS,upg}=1$ results because material properties adopted in analyzing existing constructions are generally defined through mean values, differently from new constructions where design values are adopted instead. Partial upgrades are considered acceptable in case of economical and/or practical constraints.

It is still unclear whether an upgrade can actually achieve, even unintentionally, a reduction of the initial capacity dispersion. Therefore, in the absence of more accurate estimates, in the following it will be assumed that $\beta _{LS,upg}=\beta _{LS,ass}$. The seismic target annual *LS*-exceedance frequency for upgrading existing constructions in a certain territory, described by the variation of $k_{1}$, can be evaluated as in Eq. (13):

17 $$\begin{aligned} \widetilde{\lambda }_{f,LS,upg}(\gamma _{R,LS,upg})=\min _{k_{1}}\left[ \frac{1}{\gamma _{R,LS,upg}^{\frac{k_{1}}{b}}}\cdot \lambda _{LS}\cdot \exp \left( \frac{1}{2}\frac{k_{1}^{2}}{b^{2}}\beta _{LS,ass}^{2}\right) \right] \end{aligned}$$

It should be noticed that, usually, $\beta _{LS,ass}>\beta _{LS}$, because the capacity of existing constructions is generally more dispersed. As a consequence, and because $\gamma _{R,LS,upg}\le 1$, we obtain that $\widetilde{\lambda }_{f,LS,upg}>\widetilde{\lambda }_{f,LS}$, that is to say, the target frequency of an upgrade procedure is higher than that of a design procedure, i.e., characterized by a larger risk. This is inherent to a practical assessment procedure as it depends on the adoption of mean values for the basic variables and on the likely higher dispersion in the capacity^69^.

#### Minimum of $\lambda _{f,LS}$ across a territory and $k_{1}$-bounded solution

The hazard function slope $\widetilde{k}_{1}$ in log-log coordinates corresponding to the minimum of $\lambda _{f,LS}$ is:

18 $$\begin{aligned} \frac{\partial \lambda _{f,LS}}{\partial k_{1}}=0\Rightarrow \widetilde{k}_{1}=\frac{b\cdot \ln \gamma _{R,LS}}{\beta _{LS}^{2}} \end{aligned}$$

By setting a lower bound $k_{1,min}$ and an upper bound $k_{1,max}$ within a territory, the value $\widetilde{k}_{1}$ within such range corresponding to the minimum in Eq. (13) given ($\gamma _{R,LS}$, *b*, $\beta _{LS}$) can be evaluated as:

19 $$\begin{aligned} \widetilde{k}_{1}={\left\{ \begin{array}{ll} k_{1,min} &{} \text {if }\frac{\ln \gamma _{R,LS}}{\beta _{LS}^{2}}\le k_{1,min}\\ \frac{b\cdot \ln \gamma _{R,LS}}{\beta _{LS}^{2}} &{} \text {if }k_{1,min}<\frac{\ln \gamma _{R,LS}}{\beta _{LS}^{2}}\le k_{1,max}\\ k_{1,max} &{} \text {if }k_{1,max}\le \frac{\ln \gamma _{R,LS}}{\beta _{LS}^{2}} \end{array}\right. } \end{aligned}$$

The rationale behind employing a $k_{1}$-bounded solution lies in confining the analysis to specific regions within the territory where elevated seismic hazards are prevalent, as discussed in Section “Application to Europe for peak ground accelerations” and illustrated in Fig. 9b. The proposed solution exhibits a high degree of generality, and by assigning an exceedingly large value to the parameter $k_{1,max}$, an upper unbounded condition can be achieved (similarly, by specifying $k_{1,min}$ to define the lower boundary).

**Figure 3:**
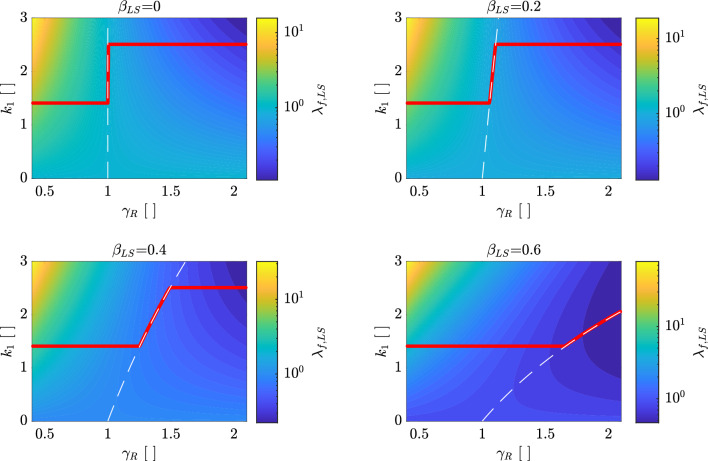
$\lambda _{f,LS}$ [Eq. (10)] for $\lambda _{LS}=1$ and $b=1$ as a function of $k_{1}$ and $\gamma _{R,LS}$ for different values of $\beta _{LS}=\left\{ 0,\,0.2,\,0.4,\,0.6\right\}$. The red line shows the location of $\widetilde{k}_{1}$ ($k_{1,min}=1.4$ and $k_{1,max}=2.5$) found numerically (or equivalently using Eq. (19). The dashed white line shows Eq. (18).

An example of $\lambda _{f,LS}$ [Eq. (10)] for $\lambda _{LS}=1$ as a function of $k_{1}$ and $\gamma _{R,LS}$ for different values of $\beta _{LS}=\left\{ 0,\,0.2,\,0.4,\,0.6\right\}$ is shown in Fig. 3 where $k_{1,min}=1.4$ and $k_{1,max}=2.5$ are adopted (this range will be discussed in Section “Hazard model”). These values are representative of the European territory as it will be discussed in Section “Application to Europe for peak ground accelerations”. $\widetilde{k}_{1}$ (corresponding to the minimum of $\lambda _{f,LS}$) is calculated and shown with a red line while Eq. (18) is shown with a dashed white line. From the figure, it can be seen that Eq. (18) is always equal to $\widetilde{k}_{1}=0$ for $\gamma _{R,LS}=1$. The solution of Eq. (18) is a vertical line for $\beta _{LS}=0$ whose inclination is reducing with increasing $\beta _{LS}$. Accordingly, for $\beta _{LS}=0$, $\widetilde{k}_{1}$ is equal to $k_{1,min}$ for $\gamma _{R,LS}<1$ and $\widetilde{k}_{1}=k_{1,max}$ for $\gamma _{R,LS}>1$. For $\beta _{LS}>0$, $\widetilde{k}_{1}$ is always equal to $k_{1,min}$ for $\gamma _{R,LS}<1$ and tending to $k_{1,max}$ for $\gamma _{R,LS}>1$.

The application of this approach to Europe for peak ground accelerations will be discussed in Section “Application to Europe for peak ground accelerations” using appropriate values of $k_{1,min}$ and $k_{1,max}$.

### Risk-based mean return period and risk-based intensity

**Figure 4:**
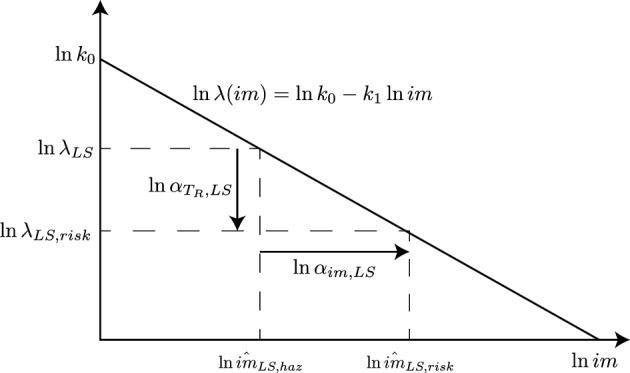
Schematic of modification factors to obtain risk-based mean return period and risk-based intensity.

If $\widetilde{\lambda }_{f,LS}$ is determined, then the risk-targeted mean return period $T_{R,LS,risk}=\lambda _{LS,risk}^{-1}$ and the corresponding median intensity used in design $\hat{im}_{LS,risk}$ can be obtained. Notice that, for design purposes, the return period is more practical than the mean annual frequency. Thus, $\lambda _{LS,risk}^{-1}$ is obtained by multiplying the corresponding uniform-hazard-based value, $\lambda _{LS}^{-1}$, by a modification factor $\alpha _{T_{R},LS}$, while $\hat{im}_{LS,risk}$ is obtained by multiplying the corresponding uniform-hazard-based value, $\hat{im}_{LS,haz}$, by a modification factor $\alpha _{im,LS}$. These two factors are represented in Fig. 4. The key basic idea of these two factors is to correct the uniform-hazard-based mean return period and intensity to achieve a prescribed risk level $\widetilde{\lambda }_{f,LS}$. In other words, the two factors modify the hazard to obtain $\widetilde{\lambda }_{f,LS}$ in Eq. (10) given the set of parameters ($k_{1}$, $\gamma _{R,LS}$, *b*, $\beta _{LS}$).

Looking at Fig. 4, $\alpha _{T_{R},LS}$ is the ratio between the hazard-based mean annual frequency $\lambda _{LS}$ and the corresponding risk-based mean annual frequency $\lambda _{LS,risk}$, found in Eq. (12), which corresponds to a certain target $\widetilde{\lambda }_{f,LS}$:

20 $$\begin{aligned} \alpha _{T_{R},LS}=\frac{\lambda _{LS}}{\lambda _{LS,risk}}=\frac{1}{\gamma _{R,LS}^{\frac{k_{1}}{b}}}\frac{\lambda _{LS}}{\widetilde{\lambda }_{f,LS}}\exp \left( \frac{1}{2}\frac{k_{1}^{2}}{b^{2}}\beta _{LS}^{2}\right) \end{aligned}$$

$\alpha _{im,LS}$ is the ratio between the median risk-targeted intensity measure, $\hat{im}_{LS,risk}$, and the corresponding hazard-based intensity measure, $\hat{im}_{LS,haz}$, both computed using Eq. (6), with the appropriate frequency:

21 $$\begin{aligned} \alpha _{im,LS}=\frac{\hat{im}_{LS,risk}}{\hat{im}_{LS,haz}}=\left( \alpha _{T_{R},LS}\frac{k_{0}}{\lambda _{LS}}\right) ^{1/k_{1}}\left( \frac{k_{0}}{\lambda _{LS}}\right) ^{-1/k_{1}}=\alpha _{T_{R},LS}^{1/k_{1}} \end{aligned}$$

Notice that Eq. (10) can be recognized within Eq. (20), so that the latter can be written as:

22 $$\begin{aligned} \alpha _{T_{R},LS}=\frac{\lambda _{f,LS}}{\widetilde{\lambda }_{f,LS}} \end{aligned}$$

which shows that, at any given site, the risk-based return period modification factor is the ratio between the *LS*-exceedance probability of a uniform-hazard-based design and the target uniform-risk *LS*-exceedance probability, taken as the minimum over that territory.

**Figure 5:**
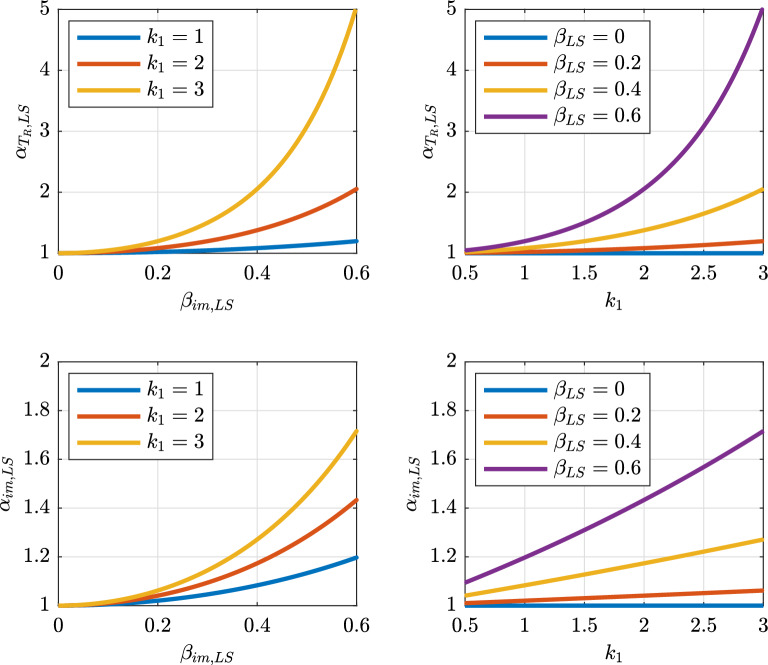
Variation of $\alpha _{T_{R},LS}$ in Eq. (20) (top) and $\alpha _{im,LS}$ in Eq. (21) (bottom) with respect to $\beta _{LS}$ (left) and $k_{1}$ (right). ($\gamma _{R,LS}=1$; $b=1$; $\lambda _{LS}=1/475\,\text{years}=0.0021$ years$^{-1}$).

The two factors do not depend on $k_{0}$ because defined as ratios. These two factors can be readily applied in any available uniform hazard model to obtain a risk-targeted hazard model. In particular, the uniform hazard model can be simply multiplied (in terms of *im* by Eq. (21) or $T_{R}$ by Eq. (20)) to obtain a risk-targeted hazard model. Figure 5 shows the variation of $\alpha _{T_{R},LS}$ and $\alpha _{im,LS}$ as a function of $k_{1}$ and $\beta _{LS}$ assuming $\gamma _{R,LS}=1$, $b=1$, $\lambda _{LS}=1/475\,\text{years}=0.0021$. Both $\alpha _{T_{R},LS}$ and $\alpha _{im,LS}$ increase with $\beta _{LS}$ and $k_{1}$. In particular, they are equal to 1 only when $\beta _{LS}=0$, thus confirming the findings reported in Section “Explanation of non-uniform risk conditions”.

## Application to Europe for peak ground accelerations

This section employs the framework presented in Sections “Seismic probability assessment formulation” and “Risk-targeted intensity measure” to calibrate the risk-targeted values for Europe and shows some examples of application of the risk-based $\alpha _{T_{R},LS}$ and $\alpha _{im,LS}$ modification factors. The target mean annual frequency $\widetilde{\lambda }_{f,LS}$, specific to seismic design, is calibrated using the procedure presented in Section “Target risk”. In order to solve Eq. (13), the fragility function properties ($\gamma _{R,LS}$, *b*, $\beta _{LS}$) of constructions code-designed to a hazard-based intensity $\hat{im}_{LS,haz}$ (i.e., corresponding to a certain $\lambda _{LS}$), and the site-hazard parameter $k_{1}$ are required. Then, the definition of the Limit States will be provided and reasonable parameters ($\gamma _{R,LS}$, $\beta _{LS}$) for new and existing constructions discussed. In the following, a hazard model for Europe and Turkey is presented. From now on, $b=1$ will be assumed. Similar considerations can be done if $b\ne 1$ and, for the sake of shortness, the details are left to the reader.

### Hazard model

The 2013 European Seismic Hazard Model (ESHM13)^70,71^ is a consistent time-independent seismic hazard model including the quantification of uncertainties for Europe and Turkey without the limits of national borders. The hazard results are available for various spectral periods (up to $10\,\text{s}$), for 6 values of $\lambda _{LS}$ ranging from $2.01\cdot 10^{-4}$ to $1.37\cdot 10^{-2}$ years$^{-1}$ (return periods ranging from 73 to 4975 years). The associated uncertainties are also given. In the following, the mean *pga* is used as *im* to evaluate $k_{0}$ and $k_{1}$ in Eq. (3). $k_{0}$ and $k_{1}$ are found by fitting Eq. (3) to the 6 couple points available (e.g., *pga* and $\lambda _{LS}$)  using a least square approach. The data are interpolated to have a constant point spacing of $5\,\text{km}$ using a Delaunay triangulation of the scattered sample points to perform interpolation^72^. The constant grid spacing allows performing spatial statistics considering each point of the grid. Only the land points are considered while the sea points are removed from the analysis. It is noteworthy that a new version of the European Seismic Hazard Model was recently released^73^.

**Figure 6:**
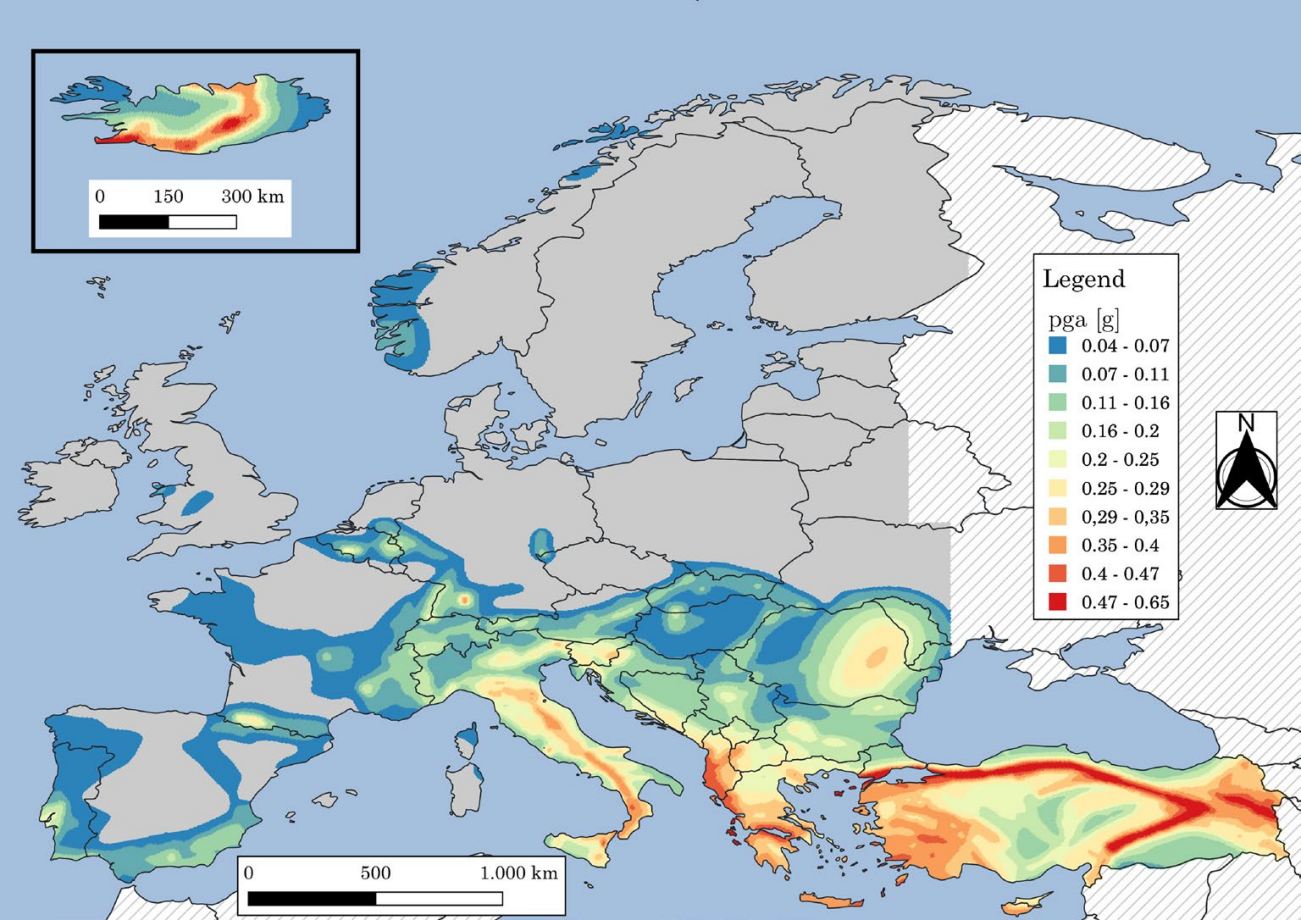
Maps for Europe and Turkey according to the 2013 European Seismic Hazard Model (ESHM13)^70,71^ of: *pga* for $\lambda _{LS}=0.0021$ years$^{-1}$ ($T_{R,LS}=475\,\text{years}$). Grey areas indicate regions where *pga* for $\lambda _{LS}=0.0021$ years$^{-1}$ ($T_{R,LS}=475\,\text{years}$) is less than $0.04\,\text{g}$. Hatching indicates regions where hazard is not provided. The map is generated using QGIS 3.16.16 (https://www.qgis.org).

The map for Europe and Turkey according to the 2013 European Seismic Hazard Model (ESHM13)^70,71^ of the *pga* for $\lambda _{LS}=0.0021$ years$^{-1}$ ($T_{R,LS}=475\,\text{years}$) is reported in Fig. 6. Only the points of the grid with non-low seismicity are considered. The low seismicity points are shown with grey areas and their definition follows EuroCode 8^5^: where the design ground acceleration *pga* for $\lambda _{LS}=0.0021$ years$^{-1}$ ($T_{R,LS}=475\,\text{years}$) is less than $0.04\,\text{g}$.  When discussing the minimum PGA considered, it is important to note that lower seismic design levels can result in over-design, as demonstrated by Gkimprixis et al.^16^. This over-design leads to lower risk, and therefore, incorporating these areas would lead to excessively conservative risk targets. The target risk for new and existing constructions (see Sections “Calibration of the seismic target risk for new constructions” and “Calibration of the seismic target risk for existing constructions”) is calculated evaluating the minimum target annual *LS*-exceedance frequency ($\widetilde{\lambda }_{f,LS}$) across the territory defined by Fig. 6.

**Figure 7:**
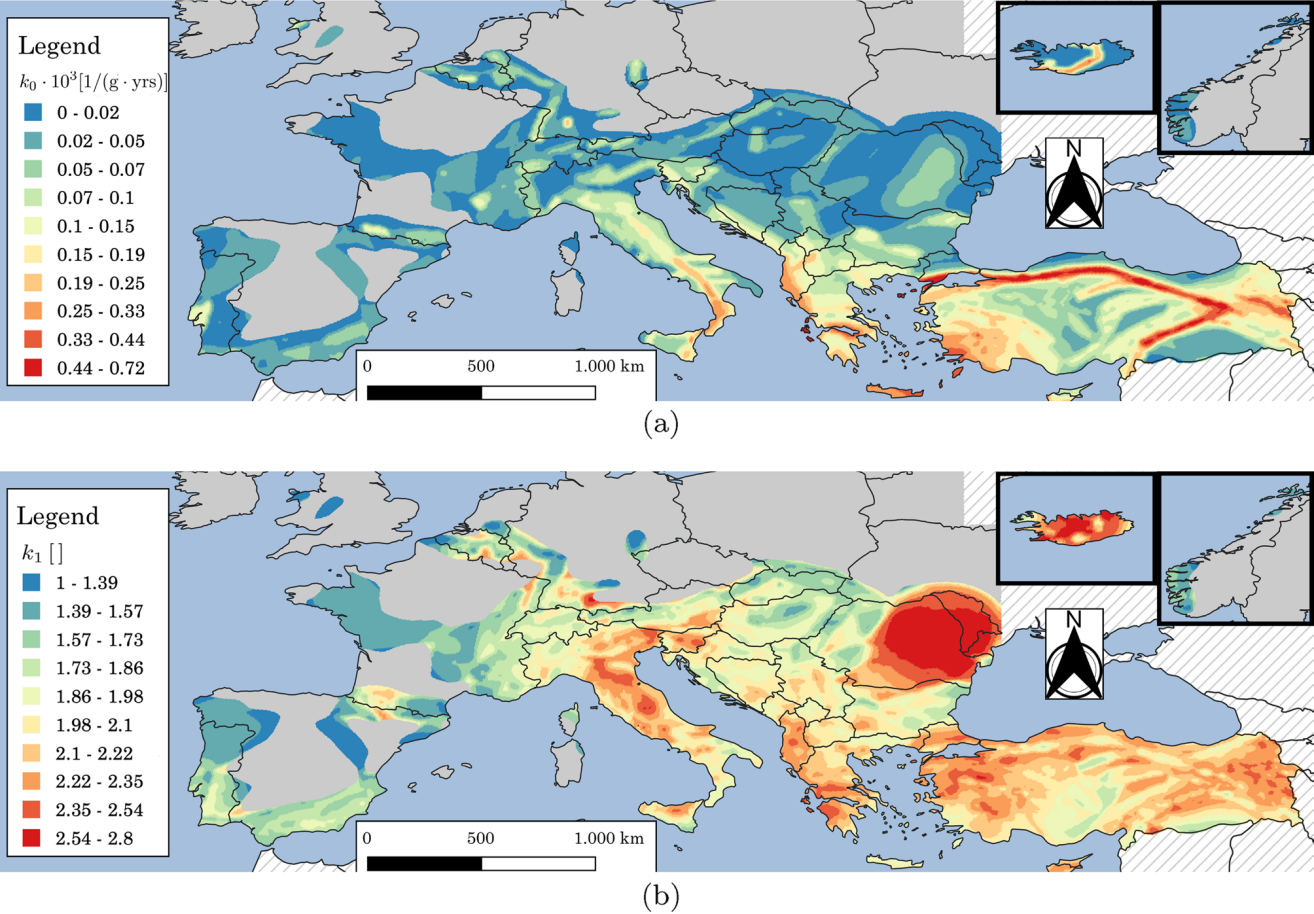
Maps for Europe and Turkey according to the 2013 European Seismic Hazard Model (ESHM13)^70,71^ of: (**a**) $k_{0}$ and (**b**) $k_{1}$ estimated according to Eq. (3) using *pga* for $\lambda _{LS}$ ranging from $2.01\cdot 10^{-4}$ to $1.37\cdot 10^{-2}$ years$^{-1}$ (return periods from 73 to 4975 years). Grey areas indicate regions where *pga* for $\lambda _{LS}=0.0021$ years$^{-1}$ ($T_{R,LS}=475\,\text{years}$) is less than $0.04\,\text{g}$. Hatching indicates regions where hazard is not provided. The map is generated using QGIS 3.16.16 (https://www.qgis.org).

**Figure 8:**
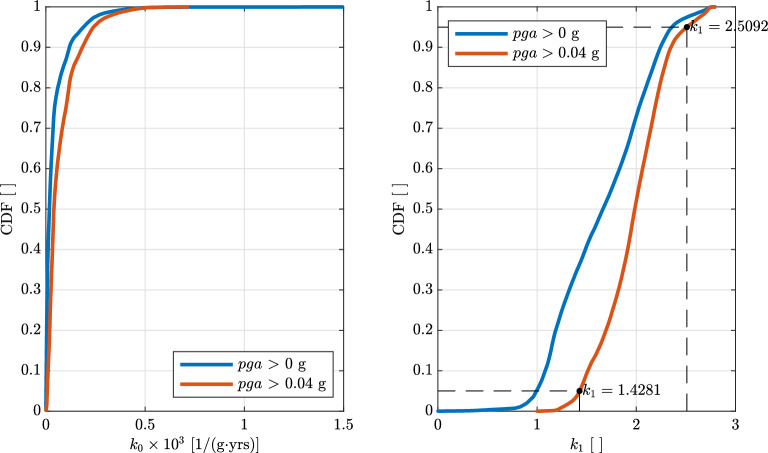
Empirical cumulative distributions of (left) $k_{0}\times 10^{3}$ and (right) $k_{1}$ using the 2013 European Seismic Hazard Model (ESHM13)^70,71^ estimated according to Eq. (3) using *pga* ranging from $2.0101\cdot 10^{-4}$ to $1.37\cdot 10^{-2}$ years$^{-1}$ (return periods from 73 to 4975 years). The two colors indicate data obtained in regions where *pga* for $\lambda _{LS}=0.0021$ years$^{-1}$ ($T_{R,LS}=475\,\text{years}$) is larger than $0\,\text{g}$ and $0.04\,\text{g}$. The two points in the right figure are the fractile of $5\%$ and $95\%$ of $k_{1}$ evaluated in regions where *pga* for $\lambda _{LS}=0.0021$ years$^{-1}$ ($T_{R,LS}=475\,\text{years}$) is larger than $0.04\,\text{g}$.

The maps for Europe and Turkey of $k_{0}$ and $k_{1}$ are reported in Fig. 7a and b, respectively. The empirical cumulative distributions of $k_{0}$ and $k_{1}$ are shown in Fig. 8 for $pga>0\,\text{g}$ (blu line) and $pga>0.04\,\text{g}$ (orange line) for $\lambda _{LS}=0.0021$ years$^{-1}$ ($T_{R,LS}=475\,\text{years}$). Only considering the last case ($pga>0.04\,\text{g}$), $k_{0}$ is in the range of 0 to around $0.8\times 10^{-3}$ while $k_{1}$ in the range of 1 to around 2.8. The $5\%$ and $95\%$ fractiles of $k_{1}$, evaluated in regions where $pga>0.04\,\text{g}$ for $\lambda _{LS}=0.0021$ years$^{-1}$ ($T_{R,LS}=475\,\text{years}$), are equal to 1.4 and 2.5. In the following, these two values will be used as $k_{1,min}$ and $k_{1,max}$, see Eq. (19). It is noteworthy that the values reported in Fig. 7b are consistent with the values commonly provided for the U.S.^9,74,75^ and previously provided for Europe using the same dataset^21^. For California and other high seismic sites with seismicity dominated by close active faults with high recurrence rates associated with tectonic plate boundaries in the U.S., $k_{1}$ typically ranges from 1.5 to 2.25^74^, which is a slightly smaller range than the $5\%$ and $95\%$ fractiles of $k_{1}$ for Europe. It is noteworthy that the values of $k_{1}$ provided in this study are slightly smaller than those provided by Gkimprixis et al.^21^. The reason behind this difference is related to the different fitting techniques employed.

**Figure 9:**
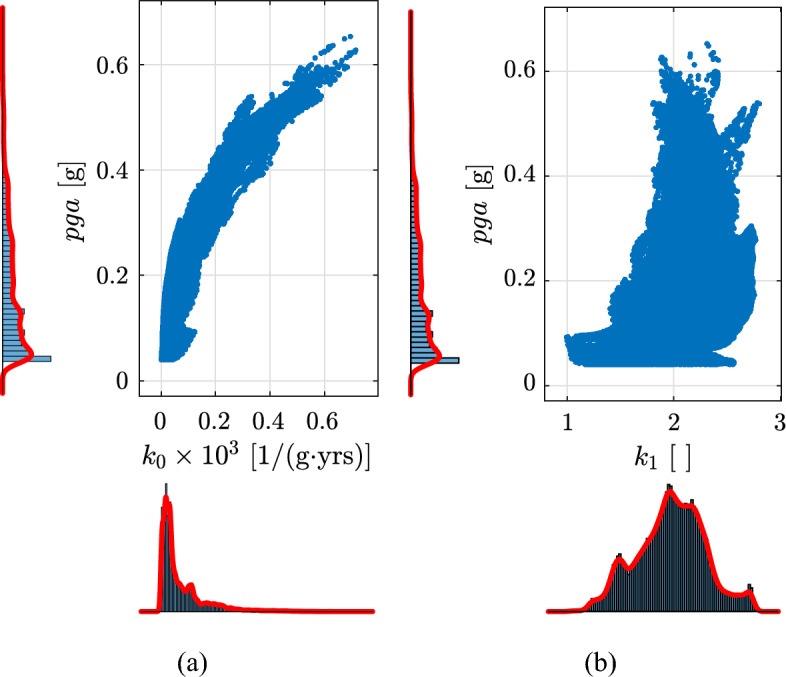
Scatter plot with marginal distributions of *pga* for $\lambda _{LS}=0.0021$ years$^{-1}$ ($T_{R,LS}=475\,\text{years}$) vs. $k_{0}\times 10^{3}$ (a) and vs. $k_{1}$ (b). Only *pga* for $\lambda _{LS}=0.0021$ years$^{-1}$ ($T_{R,LS}=475\,\text{years}$) larger than $0.04\,\text{g}$ is considered.

Figure 9 shows the scatter plots with marginal distributions of *pga* for $\lambda _{LS}=0.0021$ years$^{-1}$ ($T_{R,LS}=475\,\text{years}$) vs. $k_{0}\times 10^{3}$ and vs. $k_{1}$. From the figure, it can be seen that *pga* and $k_{0}$ are correlated with a non-linear trend with a relatively small scatter. This strong correlation results from the assumed hazard model [Eq. (3)]. On the other hand, $k_{1}$ vs. *pga* are less correlated and characterized by a large scatter. Moreover, it can be observed that assuming $k_{1,min}=1.4$ and $k_{1,max}=2.5$ only relatively low seismicity areas are not considered in the calculations (i.e., *pga* less than $0.1\,\text{g}$) and a small part of high seismicity areas. Finally, Fig. 9b can be used to check the seismicity levels of $\widetilde{k}_{1}$ in Eq. (18).

### Definition of limit states

In seismic design and assessment, the earthquake intensity used to check a certain *LS*-exceedance is obtained by fixing its mean return period $T_{R,LS}$. In this section, the latest draft of EN 1998-1-1 is considered, whereby three Limit States are considered: Damage Limitation (*DL*), Significant Damage (*SD*), and Near Collapse (*NC*). Table 1 shows, as a reference, the mean return period $T_{R,LS}$ and the annual frequency $\lambda _{LS}$ of the seismic action, with reference to the so-called Consequence Class 2 (most residential buildings, typical bridges).

**Table 1:** Mean return period $T_{R,LS}$ in years of the earthquake intensity used to check a certain Limit State (*LS*) and corresponding annual exceedance frequency $\lambda _{LS}$.

Limit state	Damage limitation	Significant damage	Near collapse
Acronym	*DL*	*SD*	*NC*
$T_{R,LS}$	60 years	475 years	1600 years
$\lambda _{LS}$	0.01667 years$^{-1}$	0.00211 years$^{-1}$	0.00625 years$^{-1}$

### Calibration of the seismic target risk for new constructions

#### Estimation of $\gamma _{R}$ and $\beta _{LS}$

In Eq. (7), the median capacity is defined as the median demand multiplied by a factor $\gamma _{R,LS}$. In a construction optimally designed according to the principles of Eurocode 0^63^ in Section 6.3.5, the design capacity is made equal to the median demand, at the *LS* of interest. Thus, $\gamma _{R,LS}$ represents the ratio of the median capacity to the design capacity, which, for lognormal distributions (see Section “Log-normality assumption for capacity and demand”), can be expressed as (Eurocode 0^63^—Table C3):

23 $$\begin{aligned} \gamma _{R,LS}=\exp (\alpha _{R}\widetilde{\beta }_{f1,LS}\widetilde{\beta }_{C,LS}) \end{aligned}$$

as function of the first order reliability method (FORM) sensitivity factor for capacity $\alpha _{R}$, the target annual reliability index $\widetilde{\beta }_{f1,LS}$, and the (target) coefficient of variation of the system global capacity $\widetilde{\beta }_{C,LS}$.

According to the best authors’ knowledge, values of $\alpha _{R}$, $\widetilde{\beta }_{f1,LS}$, and $\widetilde{\beta }_{C,LS}$ are not provided for all the *LS*s of Table 1. However, reasonable values of $\alpha _{R}$, $\widetilde{\beta }_{f1,LS}$, and $\widetilde{\beta }_{C,LS}$ based on several standards and code of practices and authors’ considerations are provided with the aim of developing an example of application of the proposed procedure. These values can be eventually fine-tuned to calibrate the outcomes. It is noteworthy that, for specific constructions, $\gamma _{R,LS}$ can be calibrated through Finite Element Method (FEM) analyses as noticed by Vamvatsikos and Cornell^76^ and as carried out by several authors, notably, Schlune et al.^77^, Cervenka^78^, Allaix et al.^79^, Belletti et al.^80^, Pimentel et al.^81^, Blomfors et al.^82^, Castaldo et al.^83^, although this is beyond the scope of this study, which is to provide a general procedure for defining seismic target risk in Europe.

The proposed values of $\alpha _{R}$, $\widetilde{\beta }_{f1,LS}$ (and corresponding $\widetilde{P}_{f1,LS}$) and $\widetilde{\beta }_{C,LS}$ are provided in Table 2. In particular, $\widetilde{\beta }_{f1,LS}$ for *NC* is taken from Table G.4 in ISO 2394^64^, for Consequence Class 3 (most residential buildings, typical bridges) and medium cost of safety measures, since for new constructions the relative costs of safety measures can be generally considered moderate^84^. $\widetilde{\beta }_{f1,LS}$ for *DL* is taken from Table E.2 in ISO 2394^64^, where the original value $\widetilde{\beta }_{f50,LS}=2.3$ for *DL* (with the subscript 50 indicating the reference period) is given for life-time and normal cost of safety measures. The corresponding annual value for normal cost $\widetilde{\beta }_{f1,LS}=2.8$ for *DL* is found by considering a life-time of $50\,\text{years}$ and converting them into $1\,\text{year}$ assuming independence period equal to 10 years. $\widetilde{\beta }_{f1,LS}$ for *SD* is assumed to be 3.8 being in the range between *DL* and *NC* and sufficiently near to *NC*.

The FORM sensitivity factor $\alpha _{R}$ is the directional cosine with respect to the capacity axis of the most probable failure point in an underlying independent standard normal space^85^. The standardized FORM factor $\alpha _{R,50}=0.8$ (Eurocode 0^63^ appendix C) has been derived considering reliability targets for a 50-year reference period^86^. However, this value cannot be applied to seismic cases, where the uncertainty in the capacity is less relevant. Therefore, $\alpha _{R,50}$ is here calibrated so as to yield a *NC*-exceedance probability consistent with the provisions of ASCE 7–16^24^, which establishes a mean annual collapse probability of $2\times 10^{-4}$ for the conterminous United States for a 2% hazard-exceedance probability in 50 years  (i.e., a 0.04% annual probability). The calibrated value of  $\alpha _{R,50}=0.42$ is such that Eq. (12) yields exactly the value $2\times10^{-4} \,{\text{when}} \,\widetilde{\lambda}_{f,LS}=0.04\% \, {\text{and}} \,{\text{with}}\, \widetilde{\beta}_{f1,LS} \, {\text{and}} \,\widetilde{\beta}_{C,LS}$ as in Table 2. Notice that the annual sensitivity factor $\alpha _{R}$ used to compute $\gamma_{R,LS}$ from Eq. (23) depends on $\alpha_{R,50}$  through the ratio between the target 50-year reliability index and the target annual reliability index, as suggested by Meinen and Steenbergen ^87^ in Section 6.3, as:

24 $$\begin{aligned} \alpha _{R}=\alpha _{R,50}\cdot \frac{\widetilde{\beta }_{f50,LS}}{\widetilde{\beta }_{f1,LS}} \end{aligned}$$

Thus, $\alpha _{R}=0.42\cdot \widetilde{\beta }_{f50,LS}/\widetilde{\beta }_{f1,LS}$ is considered and the so-obtained values are summarized in Table 2. As expected, $\alpha _{R}$ increases from *DL* to *NC* consistently with the larger uncertainties in the capacity model with respect to the demand one.

**Table 2:** Target values of $\widetilde{\beta }_{f1,LS}$, $\widetilde{P}_{f1,LS}$ and $\gamma _{R,LS}$.

	Limit State		
	*DL*	*SD*	*NC*
$\widetilde{\beta }_{f1,LS}$	2.8$^{\clubsuit }$	3.8	4.2$^{\spadesuit }$
$\widetilde{P}_{f1,LS}$ $^{*}$	$2.6\times 10^{-3}$	$6.7\times 10^{-5}$	$1.3\times 10^{-5}$
$\alpha _{R}$	0.34	0.37	0.38
$\beta _{D,LS}$	0.20	0.40	0.40
$\beta _{C,LS}$	0.35	0.45	0.45
$\beta _{LS}$	0.40	0.60	0.60
$\gamma _{R,LS}$	1.38	1.89	2.05

$^{\clubsuit }$ From ISO 2394^88^; $^{\spadesuit }$ From ISO 2394^64^. $^{*}$ $P_{f1,LS}=-\Phi \left( \beta _{f1,LS}\right) .$

The values to assign to $\beta _{LS}$, due to its effect on $\widetilde{\beta }_{f1,seis,LS}$, should be treated carefully. FEMA published the FEMA P695^55^ methodology, based on the Applied Technology Council (ATC)-63 work, that aimed at providing a rigorous basis to quantitatively determine values of the building seismic performance factors, anchoring these values on results from series of incremental dynamic analyses using nonlinear time history analyses for a large number of ground motions^89^. FEMA P695^55^ proposes a model as that in Eq. (9) to estimate the total uncertainties in collapse evaluation:

25 $$\begin{aligned} \beta _{LS}^{2}=\beta _{D,LS}^{2}+\underset{\beta _{C,LS}^{2}}{\underbrace{\beta _{DR}^{2}+\beta _{TD}^{2}+\beta _{MDL}^{2}}} \end{aligned}$$

where $\beta _{DR}$ is the design-requirements-related collapse uncertainty, $\beta _{TD}$ is the test-data-related collapse uncertainty, and $\beta _{MDL}$ is the modeling-related collapse uncertainty; each in the range of 0.1 to 0.5. $\beta _{C,LS}$ is the square root sum of these three terms. It should be highlighted that the FEMA P695^55^ model is calibrated for collapse (i.e., *NC*). However, its application to other *LS*s is proposed in the following.

$\beta _{D,LS}$ is in the range of 0.2 to 0.4^55^. $\beta _{D,LS}$ can be assumed equal to 0.2 for systems that have little, or no, period elongation and 0.4 for systems with significant period elongation. Accordingly, it can be assumed that $\beta _{D,LS}=0.20$ for *DL*, and $\beta _{D,LS}=0.40$ for *SD* and *NC*. This is related to little period elongation at *DL* and to the significant period elongation of *SD* and *NC*.

$\beta _{C,LS}$ is calculated in the range 0.17–0.87^55^. $\beta _{C,LS}$ is strongly dependent on the definition of the EDP. In this study, it is assumed $\beta _{LS}=0.40$ for *DL*, and $\beta _{LS}=0.60$ for *SD* and *NC*. Using Eq. (25), $\beta _{C,LS}$ is easily derived. $\beta _{D,LS}$, $\beta _{C,LS}$, and $\beta _{LS}$ are summarized in Table 2.

The accurate estimation of $\beta _{LS}$ involves probabilistic analyses (Monte Carlo or First-Order Second Moment (FOSM)) using an accurate distribution function for structural members properties. The value obtained for *NC* is slightly higher than that suggested by using the FOSM method, i.e., 0.4^90^. The value obtained for *NC* is also in the order of that suggested by Zareian and Krawinkler^91^ using $\beta _{D,LS}=0.4$ and $\beta _{C,LS}=0.5$ (to account for all epistemic uncertainties^92^). Similar values were also observed for design level drift limits in building codes and results of other dynamic analysis studies^93^.

It should be noted that previous studies refer to performance assessment (see Section “Calibration of the seismic target risk for existing constructions”) rather than design. Accordingly, the proposed values can be considered upper bounds of the uncertainties levels for new constructions. The values of $\beta _{LS}$ at *NC* proposed here are smaller compared with the values provided by Luco et al.^13^ which are in the range of 0.8 but similar to ASCE 7–16^24^ adopting 0.6.

In the calculation of $\gamma _{R,LS}$ using Eq. (23), the actual coefficient of variation can be considered equal to the intended one, so $\widetilde{\beta }_{C,LS}=\beta _{C,LS}$. Table 2 summarizes the values of $\gamma _{R,LS}$ found using Eq. (23) for the different *LS*. The resulting $\gamma _{R,LS}$ ranges from 1.38 for *DL* to 2.05 for *NC*. This is consistent with Eurocode 0^63^ expressly reporting that different sets of $\gamma _{R,LS}$ are associated with the various ultimate limit states. Interestingly, using the 10th-percentile collapse capacity assumed by Luco et al.^13^ for $\beta _{LS}=0.6$, the resulting $\gamma _{R,LS}$ is:

26 $$\begin{aligned} \gamma _{R,LS}=\exp (-\Phi (0.1)\beta _{LS})^{-1}=\exp (1.2816\cdot 0.6)^{-1}=2.17 \end{aligned}$$

The so-obtained $\gamma _{R,LS}$ is quite comparable with the proposed 2.05 for *NC*. Finally, it is noteworthy that the choice of the 10th-percentile collapse capacity assumed by Luco et al.^13^ can be justified by several reasons: (*i*) it was adopted from FEMA codes where they performed incremental dynamic analyses in code-conforming buildings^13^; (*ii*) Kennedy and Short^74^ observed that it minimizes the influence of $\beta _{LS}$; (*iii*) Gkimprixis et al.^21^ observed that it leads to risk-targeted ground motions that do not deviate significantly from the uniform hazard ones, irrespectively of the values of $k_{1}$ and $\beta _{LS}$.

#### Target risk

**Figure 10:**
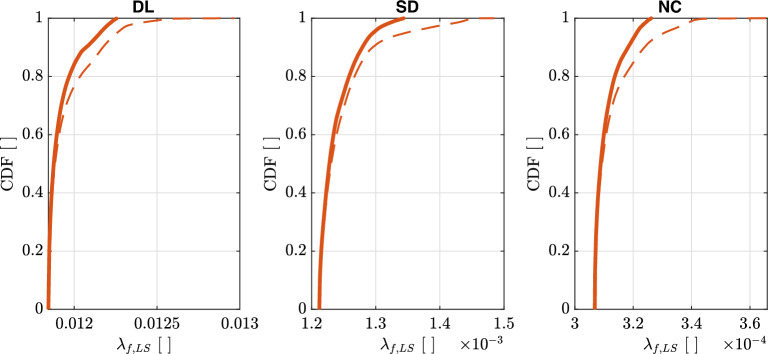
Empirical cumulative distributions of $\lambda _{f,LS}$ for the three Limit States of *DL*, *SD*, and *NC*. Thick solid lines are evaluated considering $pga\ge 0.04g$ for $\lambda _{LS}=0.0021$ years$^{-1}$ ($T_{R,LS}=475\,\text{years}$) and $1.4\le k_{1}\le 2.5$. Thin dashed lines are evaluated only considering $pga\ge 0.04g$.

Figure 10 shows the empirical cumulative distributions of $\lambda _{f,LS}$ for the three Limit States. The maps for Europe and Turkey of $\lambda _{f,LS}$ are reported in Supplementary Material. $\lambda _{f,LS}$ was calculated using Eq. (10) with the values of $k_{1}$ evaluated on the hazard model of Europe and Turkey (Figs. 7b and 8) considering $\gamma _{R,LS}$ [Eq. (23)] and $\beta _{LS}$ as reported in Section “Calibration of the seismic target risk for new constructions”. The empirical cumulative distributions of $\lambda _{f,LS}$ are calculated considering $pga\ge 0.04g$ for $\lambda _{LS}=0.0021$ years$^{-1}$ ($T_{R,LS}=475\,\text{years}$) and by also considering (thick solid lines) or not (thin dashed lines) the range $1.4\le k_{1}\le 2.5$. The latter range corresponds to the fractile of $5\%$ and $95\%$ of $k_{1}$ (Fig. 8).

From the figure, it can be observed that $\lambda _{f,LS}$ increases changing the Limit States with the following order *DL*, *SD*, and *NC*. The reason from this increase is due to the smaller values of $\lambda _{LS}$ for each *LS*s (see Table 1). Smaller values of $\lambda _{LS}$ correspond to larger values of $\lambda _{f,LS}$ because of the lower exceedance probability of $\lambda (im)$, thus higher safety levels (see Fig. 1). Similarly to $\lambda _{LS}$, an increase of $\gamma _{R,LS}$ corresponds to a lower exceedance probability of $\lambda (im)$, thus higher safety levels (see Fig. 1).

The empirical cumulative distributions of $\lambda _{f,LS}$ are used to calculate target values of $\widetilde{\lambda }_{f,LS}$ for seismic design of new constructions obtained using Eq. (13) and summarized in Table 3. The minimum over the territory of interest is calculated considering $pga\ge 0.04g$ for $\lambda _{LS}=0.0021$ years$^{-1}$ and $1.4\le k_{1}\le 2.5$, i.e., thick lines in Fig. 10. Similarly to the empirical cumulative distributions of $\lambda _{f,LS}$, $\widetilde{\lambda }_{f,LS}$ increases by changing the Limit States with the following order *DL*, *SD*, *NC*. $\widetilde{\lambda }_{f,LS}$ listed in Table 3 can also be evaluated substituting Eq. (19) in Eq. (10), to obtain (reporting *b* in the general form instead of assuming it to be 1):

27 $$\begin{aligned} \widetilde{\lambda }_{f,LS}=\frac{\lambda _{LS}}{\gamma _{R,LS}^{\frac{\widetilde{k}_{1}}{b}}}\exp \left( \frac{1}{2}\frac{\widetilde{k}_{1}^{2}}{b^{2}}\beta _{LS}^{2}\right) \end{aligned}$$

**Table 3:** Target values of $\widetilde{\lambda }_{f,LS}$ for seismic design of new constructions obtained using Eq. (27).

Limit State		
*DL*	*SD*	*NC*
$1.12\times 10^{-2}$	$1.21\times 10^{-3}$	$3.07\times 10^{-4}$

Only sites with $pga\ge 0.04g$ for $\lambda _{LS}=0.0021$ years$^{-1}$ ($T_{R,LS}=475\,\text{years}$) and $1.4\le k_{1}\le 2.5$ are considered.

### Calibration of the seismic target risk for existing constructions

#### Estimation of $\gamma _{R,LS}$ and $\beta _{LS}$

For the purpose of developing target risk values for upgrading of existing structures, the following values are taken as representative of different strategies (see Section “Target risk”): $\gamma _{R,LS,upg}=\{0.6,\,0.8,\,1.0\}$. In particular, $\gamma _{R,LS,upg}=1$ correspond to “retrofit” while 0.6 and 0.8 are “upgrade” strategies (see Fig. 2). In the absence of more accurate estimates, as commented in Section “Target risk”, uncertainties are assigned the same values as the assessed ones, which, for the purpose of this calibration, are taken equal to those adopted for new constructions, see Section “Calibration of the seismic target risk for new constructions”: as a matter of fact, fragility curves for new and existing structures differ mainly due to the $\gamma _{R,LS}$-shifted median rather than to the dispersion. It is assumed here that there is no difference in the dispersion of fragility curves for new and existing buildings (see Section “Target risk”).

#### Target risk for seismic upgrading of existing constructions

**Figure 11:**
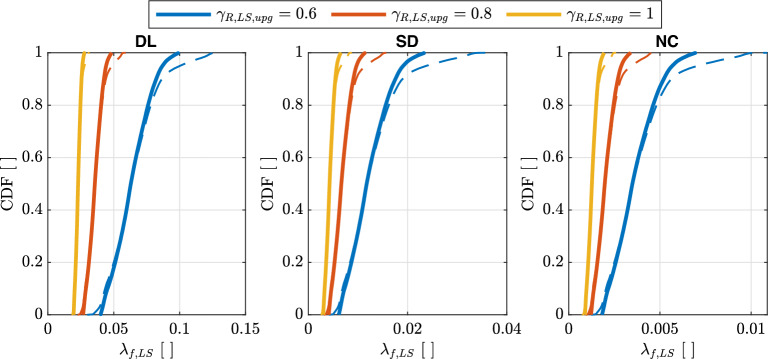
Empirical cumulative distribution of $\lambda _{f,LS}$ for the three Limit States of *DL*, *SD*, and *NC* for $\gamma _{R,LS,upg}=\{0.6,\,0.8,\,1.0\}$. Thick solid lines are evaluated considering $pga\ge 0.04g$ for $\lambda _{LS}=0.0021$ years$^{-1}$ ($T_{R,LS}=475\,\text{years}$) and $1.4\le k_{1}\le 2.5$. Thin dashed lines are evaluated only considering $pga\ge 0.04g$.

Figure 11 illustrates the empirical cumulative distributions of $\lambda _{f,LS}$ for the three Limit States and for $\gamma _{R,LS,upg}=\{0.6,\,0.8,\,1.0\}$. To provide a comprehensive analysis, all three Limit States will be taken into account in subsequent discussions. $\lambda _{f,LS}$ was calculated with the values of $k_{1}$ evaluated on the hazard model of Europe and Turkey (Figs. 7b and 8) considering $\gamma _{R,LS}$ [Eq. (23)] and $\beta _{LS}$ as reported in Section “Calibration of the seismic target risk for existing constructions”. The empirical cumulative distributions of $\lambda _{f,LS}$ are calculated considering $pga\ge 0.04\,g$ for $\lambda _{LS}=0.0021$ years$^{-1}$ ($T_{R,LS}=475\,\text{years}$) and by also considering (thick solid lines) or not (thin dashed lines) the range $1.4\le k_{1}\le 2.5$. The latter range corresponds to the fractile of $5\%$ and $95\%$ of $k_{1}$ (Fig. 8).

Similar considerations with Fig. 10 can be done. $\lambda _{f,LS}$ increases changing the Limit States with the following order *DL*, *SD*, and *NC* and an increase of $\gamma _{R,LS}$ correspond to a lower exceedance probability of $\lambda (im)$ thus higher safety level (see Fig. 1). The increase of $\gamma _{R,LS}$ induces a reduction of the variability of $\beta _{LS}$ on the territory.

Table 4 reports the target values of $\widetilde{\lambda }_{f,LS,upg}$ for $\gamma _{R,LS,upg}=\left\{ 0.6,\,0.8,\,1.0\right\}$. The minimum over the territory of interest is calculated considering $pga\ge 0.04g$ for $\lambda _{LS}=0.0021$ and $1.4\le k_{1}\le 2.5$, i.e., thick lines in Fig. 11. Similarly to the empirical cumulative distributions of $\lambda _{f,LS}$, $\widetilde{\lambda }_{f,LS,upg}$ increases by changing the Limit States with the following order *DL*, *SD*, *NC*. $\widetilde{\lambda }_{f,LS,upg}$ reported in Table 4 can also be evaluated by substituting Eq. (19) in Eq. (10) (reporting *b* in the general form instead of assuming it to be 1):

28 $$\begin{aligned} \widetilde{\lambda }_{f,LS,upg}=\frac{\lambda _{LS}}{\gamma _{R,LS,upg}^{\frac{\widetilde{k}_{1}}{b}}}\exp \left( \frac{1}{2}\frac{\widetilde{k}_{1}^{2}}{b^{2}}\beta _{LS,ass}^{2}\right) \end{aligned}$$

**Table 4:** Target values of $\widetilde{\lambda }_{f,LS,upg}$ for three different seismic upgrading strategies ($\gamma _{R,LS,upg}=\left\{ 0.6,\,0.8,\,1.0\right\}$), obtained using Eq. (28).

$\gamma _{R,LS,upg}$	Limit State		
	*DL*	*SD*	*NC*
0.6	$4.0\times 10^{-2}$	$6.1\times10^{-3}$	$1.8\times 10^{-3}$
0.8	$2.7\times 10^{-2}$	$4.1\times 10^{-3}$	$1.2\times 10^{-3}$
1.0	$1.9\times 10^{-2}$	$3.0\times 10^{-3}$	$8.9\times 10^{-4}$

Only sites with $pga\ge 0.04g$ for $\lambda _{LS}=0.0021$ years$^{-1}$ ($T_{R,LS}=475\,\text{years}$) and $1.4\le k_{1}\le 2.5$ are considered.

### Risk-based intensity and mean return period modification factors

#### New constructions

The final expression for the risk-based modification factor in Eq. (20) can be obtained by replacing Eqs. (23) and (27) (reporting *b* in the general form instead of assuming it to be 1):

29 $$\begin{aligned} \alpha _{T_{R},LS}(k_{1})=\frac{1}{\gamma _{R,LS}^{\frac{k_{1}-\widetilde{k}_{1}}{b}}}\exp \left\{ \frac{1}{2}\frac{k_{1}^{2}-\widetilde{k}_{1}^{2}}{b^{2}}\beta _{LS}^{2}\right\} \end{aligned}$$

while, according to Eq. (21), $\alpha _{im,LS}=\alpha _{T_{R},LS}^{1/k_{1}}$.

**Figure 12:**
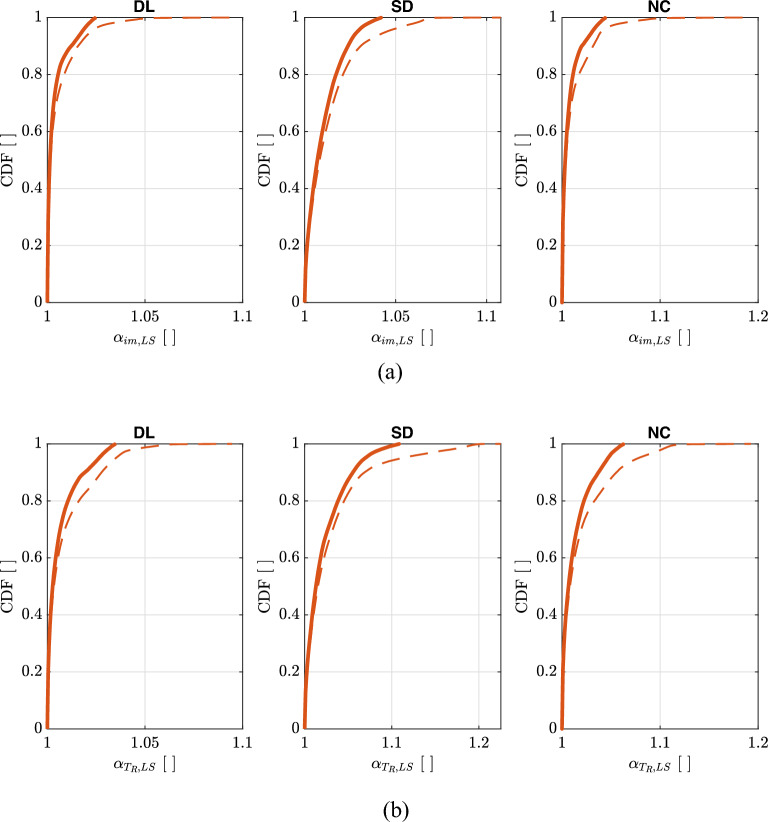
Empirical cumulative distributions of $\alpha _{T_{R},LS}$ (**a**) and $\alpha _{im,LS}$ (**b**) for the three Limit States of *DL*, *SD*, *NC*. Thick solid lines are evaluated considering $pga\ge 0.04g$ for $\lambda _{LS}=0.0021$ years$^{-1}$ ($T_{R,LS}=475\,\text{years}$) and $1.4\le k_{1}\le 2.5$. Thin dashed lines are evaluated only considering $pga\ge 0.04g$.

Figure 12 shows the empirical cumulative distributions of $\alpha _{T_{R},LS}$ and $\alpha _{im,LS}$ for the three Limit States. In both cases, $\widetilde{\lambda }_{f,LS}$ values reported in Table 3 are used.

**Figure 13:**
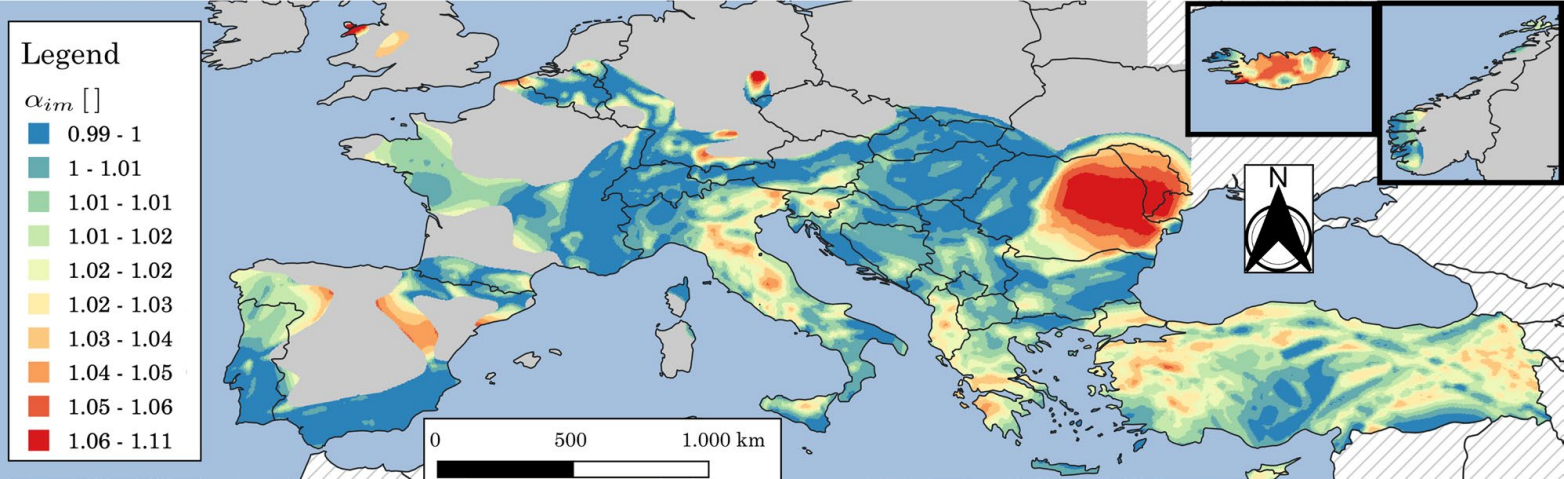
Map for Europe and Turkey of $\alpha _{im,LS}$ for *SD* design. Grey areas indicate regions where *pga* for $\lambda _{LS}=0.0021$ years$^{-1}$ ($T_{R,LS}=475\,\text{years}$) is less than $0.04\,\text{g}$. Hatching indicates regions where the hazard is not provided. The map is generated using QGIS 3.16.16 (https://www.qgis.org).

**Figure 14:**
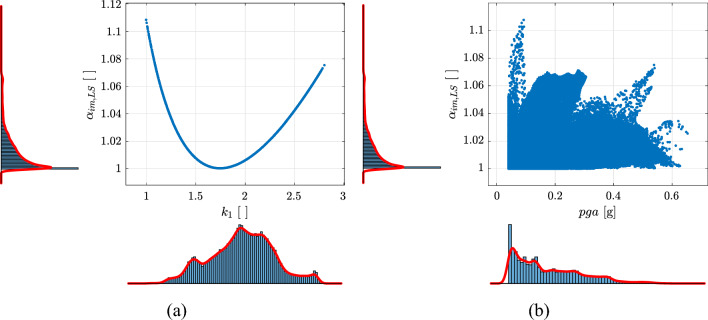
Scatter plot with marginal distributions of $\alpha _{im,LS}$ vs. $k_{1}$ (**a**) and $\alpha _{im,LS}$ vs. *pga* (**b**) for *SD* design. The points are evaluated only considering $pga\ge 0.04g$ for $\lambda _{LS}=0.0021$ years$^{-1}$ ($T_{R,LS}=475\,\text{years}$).

As an example, Fig. 13 shows the map for Europe and Turkey of $\alpha _{im,LS}$ for *SD* design. It can be observed that the maximum values of $\alpha _{im,LS}$ are located in regions where $k_{1}$ is very high (more frequent) or very low (see Figs. 7b and 14a). Finally, Fig. 14b shows the scatter plot with marginal distributions of $\alpha _{im,LS}$ vs. *pga* for *SD* design (same case of Fig. 13) where it can be seen that for new constructions the majority of the seismic areas (large values of *pga*) are characterized by relatively low risk-based intensity modification factors. This can be also qualitatively observed by comparing Figs. 6 and 13.

#### Existing constructions

The final expression for the risk-based modification factor in Eq. (20) can be obtained by replacing Eq. (17) and $\gamma _{R,LS}=\gamma _{R,LS,upg}$ (reporting *b* in the general form instead of assuming it to be 1):

30 $$\begin{aligned} \alpha _{T_{R},LS,upg}(k_{1})=\frac{1}{\gamma _{R,LS,upg}^{\frac{k_{1}-\widetilde{k}_{1}}{b}}}\exp \left\{ \frac{1}{2}\frac{k_{1}^{2}-\widetilde{k}_{1}^{2}}{b^{2}}\beta _{LS}^{2}\right\} \end{aligned}$$

while, according to Eq. (21), $\alpha _{im,LS,upg}=\alpha _{T_{R},LS,upg}^{1/k_{1}}$.

**Figure 15:**
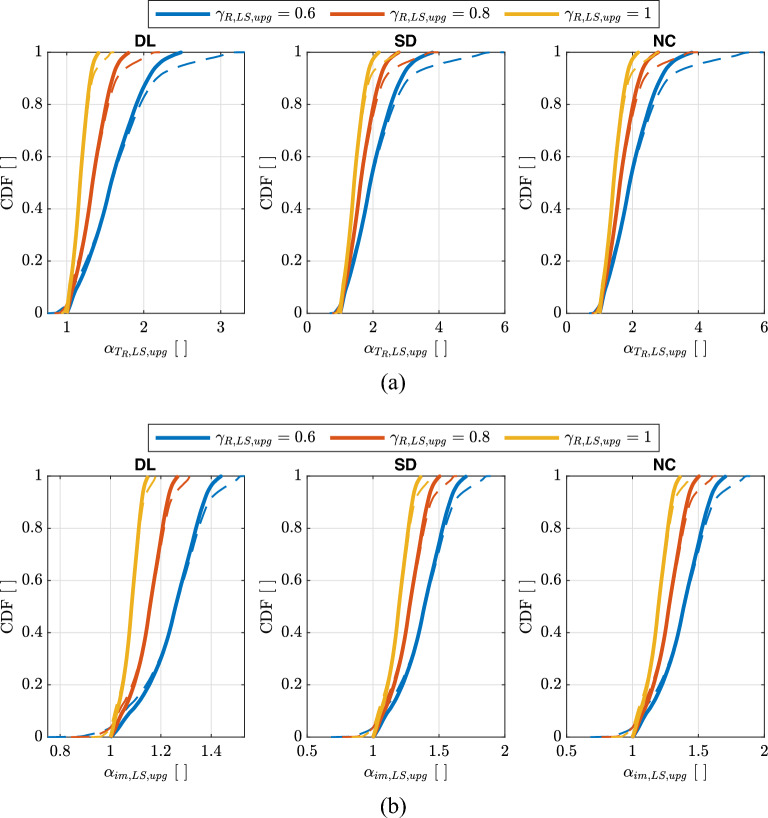
Empirical cumulative distributions of $\alpha _{T_{R},LS,upg}$ (**a**) and $\alpha _{im,LS,upg}$ (**b**) for the three Limit States of *DL*, *SD*, *NC* and for three different upgrading targets ($\gamma _{R,LS,upg}=0.6,0.8,1.0$). Thick solid lines are evaluated considering $pga\ge 0.04g$ for $\lambda _{LS}=0.0021$ years$^{-1}$ ($T_{R,LS}=475\,\text{years}$) and $1.4\le k_{1}\le 2.5$. Thin dashed lines are evaluated only considering $pga\ge 0.04g$ for $\lambda _{LS}=0.0021$ years$^{-1}$ ($T_{R,LS}=475\,\text{years}$).

Figure 15 shows the empirical cumulative distributions of $\alpha _{T_{R},LS,upg}$ and $\alpha _{im,LS,upg}$ for the three Limit States and for three different upgrading targets ($\gamma _{R,LS,upg}=0.6,0.8,1.0$). In both cases, $\widetilde{\lambda }_{f,LS,upg}$ values reported in Table 4 are used.

**Figure 16:**
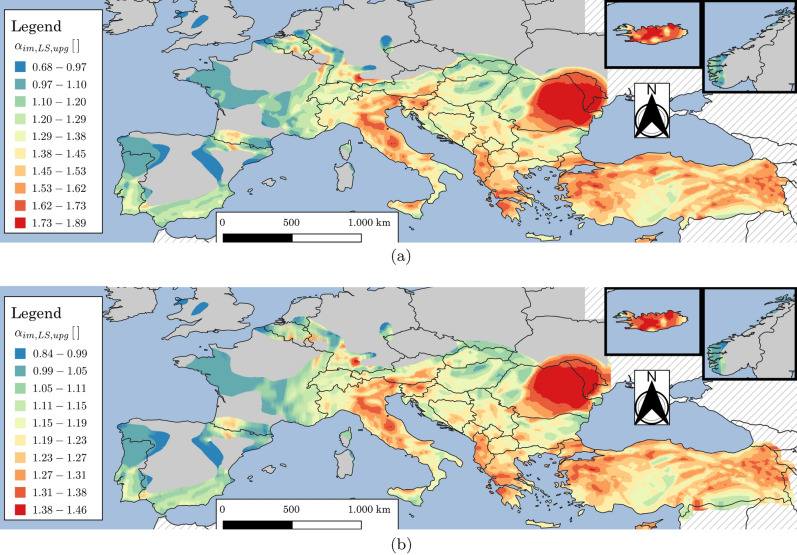
Map for Europe and Turkey of $\alpha _{im,LS,upg}$ for either *SD*-upgrading with target $\gamma _{R,LS,upg}=0.6$ (**a**) or *SD*-retrofit with target $\gamma _{R,LS,upg}=1$ (**b**) of existing constructions. Grey areas indicate regions where *pga* for $\lambda _{LS}=0.0021$ years$^{-1}$ ($T_{R,LS}=475\,\text{years}$) is less than $0.04\,\text{g}$. Hatching indicates regions where the hazard is not provided. The map is generated using QGIS 3.16.16 (https://www.qgis.org).

**Figure 17:**
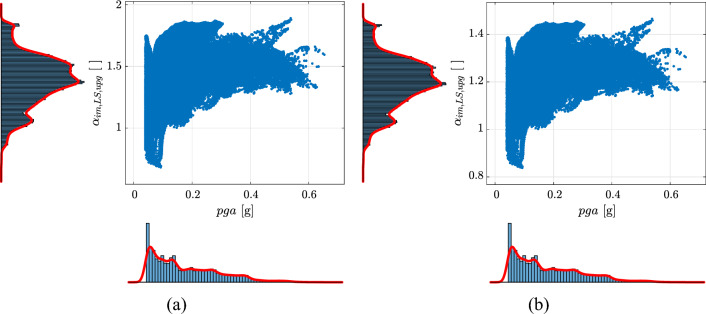
Scatter plot with marginal distributions of $\alpha _{im,LS,upg}$ vs. *pga* for $\lambda _{LS}=0.0021$ years$^{-1}$ ($T_{R,LS}=475\,\text{years}$) for either *SD*-upgrading with target $\gamma _{R,LS,upg}=0.6$ (**a**) or *SD*-retrofit with target $\gamma _{R,LS,upg}=1$ (**b**) of existing constructions. The points are evaluated only considering $pga\ge 0.04g$ for $\lambda _{LS}=0.0021$ years$^{-1}$ ($T_{R,LS}=475\,\text{years}$).

As an example, Fig. 16 shows the map for Europe and Turkey of $\alpha _{im,LS,upg}$ for *SD*-upgrading with target $\gamma _{R,LS,upg}=0.6$ or *SD*-retrofit with target $\gamma _{R,LS,upg}=1$. Finally, Fig. 17 shows the scatter plot with marginal distributions of $\alpha _{im,LS,upg}$ vs. *pga* or *SD*-upgrading with target $\gamma _{R,LS,upg}=0.6$ or *SD*-retrofit with target $\gamma _{R,LS,upg}=1$ (same case of Fig. 16). For existing constructions, the results reported herein show that for large areas of Europe the design peak ground acceleration for 475-year return period requires significant correction to achieve risk-targeted conditions. Furthermore, it has been noted that in regions with low seismic hazard (i.e., $pga\le 0.1\,\text{g}$ for $\lambda _{LS}=0.0021$ years$^{-1}$), there is a reduction in the risk targeted design values of *pga* (represented by smaller values of $\alpha _{im,LS,upg}$) due to the assumption of $k_{1,min}=1.4$ adopted in the specification of the target risk level. From Fig. 9b, it can be seen that areas with $k_{1}\le 1.4$ are localized in low seismic areas. It should be noticed that $\gamma _{R,upg}$ is acting as a scaling factor in Eq. (30) therefore results reported for $\gamma _{R,LS,upg}=0.6$ and $\gamma _{R,LS,upg}=1$ are simply scaled as well visible in Figs. 16 and 17.

## Conclusions

A contribution towards the development of risk-targeted seismic hazard models for Europe is presented in this paper, using a unified probability-based formulation that applies to, both, design and assessment/strengthening. Almost all seismic codes worldwide determine the seismic action, for both design and assessment/strengthening, based on uniform-hazard seismic maps corresponding to different hazard-exceedance probabilities. It is widely known—yet increasingly less accepted - that this approach results in non-uniform risk across a territory, which implies that designs and assessments, performed in scrupulous compliance with the code, attain different *LS*-exceedance probabilities at different sites.

Since it would be inconceivable to change all hazard-based approaches, this study proposes a possible solution to circumvent this problem and offers a practical contribution: risk-based modification factors are introduced, which multiply either the design hazard intensity, or, equivalently, the corresponding design return period. The framework is independent of the chosen hazard-based intensity measure, be it the commonly used peak ground acceleration or any other measure. These modification factors can be readily implemented in current codes to obtain risk-targeted design actions with equal *LS*-exceedance probability across a territory, without the need of changing the current hazard-based maps. It also has the twofold advantage of facilitating their application in code-based seismic engineering design practices and, at the same time, of culturally preparing the engineering community to adopt more refined risk-targeted design approaches in the future. In a sense, it can be considered as an intermediate step between the current hazard-based situation and risk-targeted design methods, which are currently still not mature enough to be accepted by the engineering community, especially for what regards assessment and strengthening.

A crucial step in developing the framework is the introduction of a factor that shifts the median capacity of the log-normal capacity. Such factor takes into account the maintainable consideration that, in actual structures, the median capacity differs from the corresponding code hazard-based demand, due either to intentional (from design) over-capacity or to undesired (e.g., in existing constructions) under-capacity. An even more crucial step is the calibration of such factor in the case of design, as explained in Section “Calibration of the seismic target risk for new constructions”. On the other hand, in the case of upgrading existing constructions, such factor is more simply defined, since it stems from the target intensity consequential to the chosen upgrading strategy.

The developed framework is applied to obtain a risk-targeted seismic hazard model for Europe, encompassing both new design and upgrading of existing constructions, based on a linear model in log-log coordinates of the hazard, under the assumption of log-normal capacity and demand. The results shown here highlight that in large areas of Europe the design peak ground acceleration for 475-year return period only requires a slight adjustment to achieve the proposed seismic risk target throughout Europe. The seismic intensities for upgrading existing constructions should be instead significantly corrected. One should inevitably concur that, while the obtained values for the modification factor may certainly be further refined, it is nonetheless evident that the current hazard-based approach proves to be less than impeccable when assessing and upgrading existing constructions. Further research should be done to provide a more refined calibration of the parameters of the structure (fragility curve) to reduce the bias between model and real structures.

## Supplementary Information

Supplementary Information.

## Data Availability

The datasets used and/or analyzed during the current study available from the corresponding author (C. Demartino) on reasonable request.

## List of symbols

$\alpha _{im,LS,upg}$: Risk-based mean intensity measure modification factor for upgrade for a certain *LS*
$\alpha _{im,LS}$: Risk-based mean intensity measure modification factor for a certain *LS*
$\alpha _{R,50}$: FORM sensitivity factor for capacity for $V_R=50$ years
$\alpha _{R}$: FORM sensitivity factor for capacity for $V_R=1$ year
$\alpha _{T_{R},LS,upg}$: Risk-based mean return period modification factor for upgrade for a certain *LS*
$\alpha _{T_{R},LS}$: Risk-based mean return period modification factor for a certain *LS*
$\beta _{C,LS}$: Log-standard deviation of the EDP capacity for a certain *LS*
$\beta _{D,LS}$: Log-standard deviation of the EDP demand for a certain *LS*
$\beta _{DR}$: Design-requirements-related collapse uncertainty
$\beta _{LS,ass}$: Log-standard deviation of the fragility curve for the assessed existing construction for a certain *LS*
$\beta _{LS,upg}$: Log-standard deviation of the fragility curve for the upgraded existing construction for a certain *LS*
$\beta _{LS}$: Log-standard deviation of the safety margin in terms of natural logarithms $\ln D-\ln C$ for a certain *LS*
$\beta _{MDL}$: Modeling-related collapse uncertainty
$\beta _{TD}$: Test-data-related collapse uncertainty
$\Delta \gamma _{R,LS}$: Factor representing the increase of the seismic median capacity for upgrade for a certain *LS*
$\gamma _{R,LS,ass}$: Factor representing the ratio between the seismic median capacity of the existing construction and the seismic median *EDP* corresponding to the median hazard-based seismic design intensity $\hat{im}_{d,LS,haz}$ for a certain *LS*
$\gamma _{R,LS,upg}$: Factor representing the ratio between the seismic median capacity of the existing construction after upgrade and the seismic median *EDP* corresponding to the median hazard-based seismic design intensity $\hat{im}_{d,LS,haz}$ for a certain *LS*
$\gamma _{R,LS}$: Factor accounts either for over-capacity or under-capacity with respect to the code hazard-based median EDP demand pertaining to a certain *LS*, see Eq. (7)
$\hat{C}_{LS,ass}$: Assessed seismic median capacity of existing constructions for a certain *LS*
$\hat{C}_{LS,upg}$: Upgraded seismic median capacity of existing constructions for a certain *LS*
$\hat{C}_{LS}$: Median *EDP* capacity for a certain *LS*
$\hat{C}_{LS}$: Median capacity for a certain *LS*
$\hat{D}(im)$: Median of the *EDP* demand conditioned on the value of *im*
$\hat{im}_{LS,haz}$: Mean intensity measure corresponding to $\lambda _{LS}$, see Eq. (6)
$\hat{im}_{LS,risk}$: Mean risk-targeted annual intensity measure for seismic design for a certain *LS*
$\lambda$: Frequency of exceedance
$$\lambda (im)$$: Hazard function
$\lambda _{f,LS}$: Mean annual exceedance frequency of a specified Limit State (*LS*)
$\lambda _{LS,ass}$: Asssessed mean risk annual frequency for existing constructions for a certain *LS*
$\lambda _{LS,risk}$: Risk-targeted mean annual frequency of hazard exceedance for a certain *LS*
$\lambda _{LS}$: Annual exceedance frequency $\lambda _{LS}$ of the hazard-based design intensity $\hat{im}_{d,haz,LS}$ for a certain *LS*
$\textbf{x}$: System properties vector
$\Omega _{im}$: Domain of *im*
$\Phi [\cdot ]$: Standard normal cumulative distribution
$\widetilde{\beta }_{C,LS}$: Coefficient of variation of the system global capacity for a certain *LS* for $V_R=1$ year
$\widetilde{\beta }_{f1,LS}$: Target annual reliability index for a certain *LS* for $V_R=1$ year
$\widetilde{\beta }_{f50,LS}$: Target annual reliability index for a certain *LS* for $V_R=50$ years
$\widetilde{\lambda }_{f,LS,ass}$: Mean risk-targeted annual frequency for seismic design for a certain *LS* for assessed constructions
$\widetilde{\lambda }_{f,LS,upg}$: Mean risk-targeted annual frequency for seismic design for a certain *LS* for upgraded constructions
$\widetilde{\lambda }_{f1,LS,seis}=\widetilde{\lambda }_{f,LS}$: Mean risk-targeted annual frequency for seismic design for a certain *LS*
$\widetilde{k}_{1}$: Target hazard function slope (minimum across the territory)
$\widetilde{P}_{f1,LS,seis}$: Mean seismic design target (acceptable) *LS*-exceedance probability for $V_R=1$ (annual values)
$\widetilde{P}_{f}$: Annual mean target (acceptable) *LS*-exceedance probability
*a*: Constant to be determined through numerical nonlinear analyses, see Eq. (4)
*b*: Constant to be determined through numerical nonlinear analyses, see Eq. (4)
$C_{LS}$: Capacity of the system at the specified *LS*
*D*: Demand on the system
*EDP*: Engineering demand parameter
$F_{LS}(im)$: Fragility function relevant to the *LS* of interest, see Eq. (2)
*im*: Intensity measure
$k_0$: Seismic hazard site-specific purely numerical constant, see Eq. (3)
$k_1$: Seismic hazard site-specific purely numerical constant, see Eq. (3)
$k_{1,max}$: Upper bound of $\widetilde{k}_{1}$, see Eq. (19)
$k_{1,min}$: Lower bound of $\widetilde{k}_{1}$, see Eq. (19)
*LS*: Limit state
*P*: Probability of exceedance
$P_{f1,LS}$: Annual mean *LS*-exceedance probability
$P_{V_{R},LS}$: Hazard-exceedance probability for a certain *LS*
*pga*: Peak ground acceleration
$T_{R,LS,risk}$: Risk-targeted mean return period for a certain *LS*
$T_{R,LS}$: Return period of the design seismic action for a certain *LS*
$$V_R$$: Reference period
