# Supplementary material for: Towards risk-targeted seismic hazard models for Europe
Source: Sci Rep. 2023 Jul 3;13:10717. doi: 10.1038/s41598-023-36947-y (PMC10318052; doi:10.1038/s41598-023-36947-y)
Supplement: Supplementary file 1 — Supplementary Information. [file 41598_2023_36947_MOESM1_ESM.pdf]

A $\lambda_{f,LS}$ for new constructions

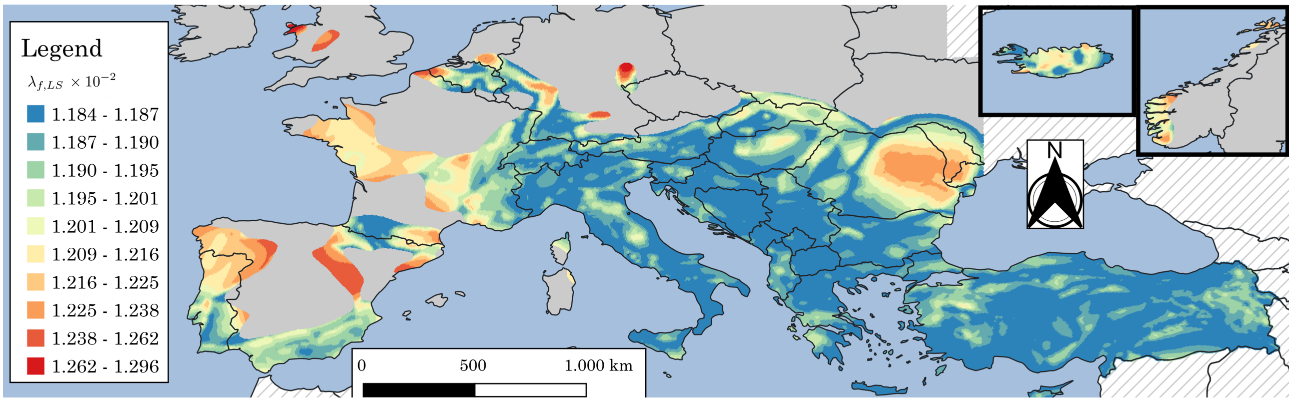

(a)

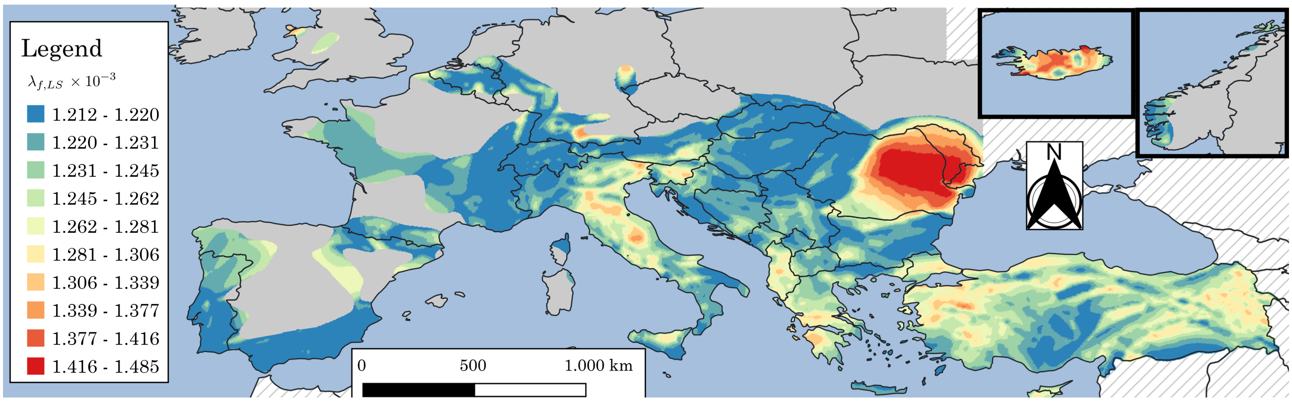

(b)

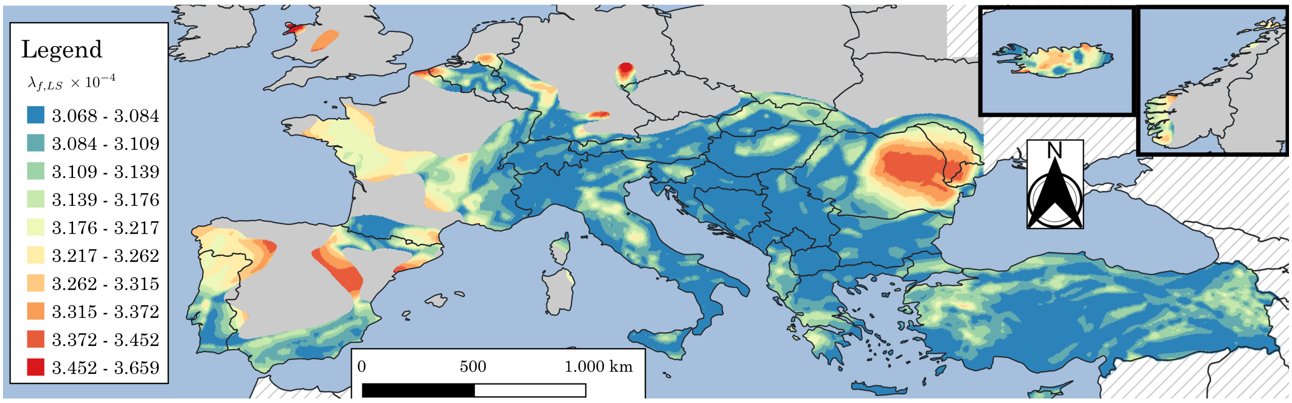

(c)

Figure 18: Map for Europe and Turkey of $\lambda_{f,LS}$ for the three Limit States of *DL* (a), *SD* (a), and *NC* (a). The map is generated using QGIS 3.16.16 (https://www.qgis.org).
